# Type I interferons induced upon respiratory viral infection impair lung metastatic initiation

**DOI:** 10.1073/pnas.2412919123

**Published:** 2026-04-17

**Authors:** Ana Farias, Victoria L. Bridgeman, Felipe S. Rodrigues, Franz Puttur, Amber Owen, Stefanie Ruhland, Rute M. M. Ferreira, Matthias Mack, Ilaria Malanchi, Cecilia Johansson

**Affiliations:** ^a^Respiratory Infections Section, National Heart and Lung Institute, Imperial College London, London W12 0NN, United Kingdom; ^b^Tumor-Host Interaction Laboratory, The Francis Crick Institute, London NW1 1AT, United Kingdom; ^c^Inflammation Repair and Development, National Heart and Lung Institute, Imperial College London, London W12 0NN, United Kingdom; ^d^Department of Nephrology, University Hospital Regensburg, Regensburg 93053, Germany

**Keywords:** virus infection, lung metastases, immune cells, epithelial cells

## Abstract

Metastatic breast cancer accounts for 7% of cancer-related deaths, with the lungs being a common site of cancer spread. In parallel, lower respiratory tract infections, including those caused by respiratory syncytial virus (RSV), remain a common cause of morbidity and mortality worldwide. Acute viral respiratory infections induce marked changes in the lung. However, how these changes influence metastasis initiation and cancer progression remains unclear. Using breast cancer and other cancer cell types in an experimental lung metastasis model, we show that RSV infection impairs tumor cell seeding and early growth in the lung, resulting in fewer metastatic nodules. We demonstrate that restriction of metastatic spread is due to alterations in the lung environment mediated by RSV-induced type I interferons (IFNs). Consistent with this idea, intranasal administration of recombinant IFN-α is sufficient to recapitulate the anti-metastatic effect of RSV infection. Using single cell RNA sequencing supported by in vivo and ex vivo validation, we show that IFN-α influences interactions between epithelial/endothelial cells and cancer cells. Furthermore, both RSV infection and IFN-α administration trigger marked local and systemic upregulation of Galectin-9, an IFN-inducible protein associated with acute respiratory infection in humans. Treatment of cancer cells with Galectin-9 alone is sufficient to restrict metastatic seeding. Altogether, our results suggest that type I IFNs induced by respiratory virus infection render the lungs less permissive to cancer cell seeding and consequently interfere with the ability of tumor cells to successfully initiate metastatic colonization.

## Significance

The lungs are a metastatic site for cancers such as breast cancer. In addition, the lungs are constantly exposed to viruses, such as coronavirus, respiratory syncytial virus (RSV), and influenza virus. Thus, breast cancer and respiratory virus infection are likely to co-occur, but their interplay remains unclear. We show that type I interferons (IFNs), induced upon viral infection impair metastatic seeding of experimental lung metastases. This occurs via IFNs acting on lung epithelial and endothelial cells, which become less supportive of early tumor cell colonization and proliferation. These findings indicate that viral infections and type I IFNs can alter the lung environment and impair metastatic initiation, which could be explored to improve future cancer treatments.

The lungs are anatomically positioned at the body–environmental interface where they play an essential role in gas exchange. The constant flow of air into the lower airways exposes the lung cells to microbes, allergens, noxious gases, and pollution particles. To prevent overexuberant immune response, the lung environment has a high threshold for immune cell activation (1). This may help provide a niche for circulating cancer cells to successfully initiate metastatic growth making the lungs the second most frequent site of metastasis for many malignancies, including colorectal cancer, head and neck cancer, breast cancer, and melanoma (2). According to the World Health Organization, cancer is a leading cause of death globally (3), with invasive breast cancer being the leading cause of cancer deaths among women (4).

Lower respiratory tract infections caused by both viral and bacterial pathogens are associated with high morbidity and mortality rates worldwide (5). Respiratory viruses, such as coronavirus, influenza virus, and respiratory syncytial virus (RSV), commonly cause infections of the upper respiratory tract that can progress to the lower tract and result in severe bronchiolitis or pneumonia (6). Seasonal vaccines for influenza virus and coronavirus are available worldwide for high-risk populations, including the immunosuppressed and the elderly, as well as recently approved vaccines against RSV (7). RSV infection is often associated with childhood bronchiolitis. However, the elderly and immunocompromised are at high risk of developing severe disease and show increased mortality rates (8, 9). Furthermore, RSV infections in older adults result in increased admissions to intensive care units compared to influenza virus infections (10).

Lung metastases and lower respiratory infections both impact the lung environment. They can co-occur, yet the interplay between them remains largely unexplored. An association between pneumonia and bronchiolitis and higher risk of lung cancer has been suggested (11, 12) and a recent study linked respiratory viral infections to the reactivation of dormant breast cancer cells in the lungs (13). However, many epidemiological findings have been based on self-reported pneumonia and primary lung cancer and do not consider the timing, number, and types of infections or cancer stage. Therefore, how changes induced in the lungs by viral infections, for example SARS-CoV-2 (14, 15), and lung malignancies impact the outcome of these diseases is an important research area.

Many respiratory viruses infect lung epithelial cells and quickly induce a pro-inflammatory response that results in the activation of resident cells and the recruitment of innate immune cells (16, 17). This innate immune response limits viral replication and orchestrates adaptive immunity, generating cytotoxic CD8^+^ T cells and antibodies that are important for viral clearance and for protection against subsequent infections (18). These immune responses change the lung environment and potentially influence lung cancer initiation, progression, and metastatic spread from primary tumors to the lungs. Cuff et al., found that influenza virus infection promotes experimental lung metastasis in the B16 melanoma model via activation of non-tumor-specific CD8^+^ T cells (19). In contrast, Newman et al., reported that influenza virus infection triggers a potent immune response that reduces B16 lung metastases (20). These conflicting results, despite using the same cancer cell model, may reflect the timing of infection relative to tumor cell injection and highlight the temporal complexity of disease interactions.

Type I interferons (IFNs) comprise of IFN-β and IFN-α subtypes and are important antiviral cytokines (17). During RSV infection, alveolar macrophages (AMs) are the main producers of type I IFNs, responding to the virus via mitochondrial antiviral signaling protein-coupled retinoic acid-inducible gene I-like receptors (21). Type I IFNs inhibit viral replication but are also involved in the recruitment of antiviral monocytes and activation of immune cells, which together control infection and disease severity (16, 21). Due to the ability of type I IFNs to modulate innate immune responses and to efficiently orchestrate the adaptive immune responses, their role in cancer immunosurveillance and their antitumoral and pro-tumoral effects have been extensively studied (22–26). However, the role of type I IFNs induced by respiratory viral infections in metastasis initiation and progression remains unclear.

We hypothesized that the early type I IFN response to respiratory virus infection may influence the ability of breast cacner cells to seed and grow in the lungs. We show that RSV infection reduces lung seeding and metastatic growth of cancer cells, resulting in lower numbers of metastatic nodules. Moreover, we demonstrate that this reduction is due to type I IFNs, which markedly alter the lung environment, generating less supportive conditions for cancer cell colonization and early metastatic growth. Our findings indicate that acute IFN responses to virus infections can rapidly remodel the lung environment to make it less supportive for cancer metastatic spread.

## Results

### RSV Infection Reduces the Number of Metastatic Nodules in the Lungs.

**Fig. 1.**
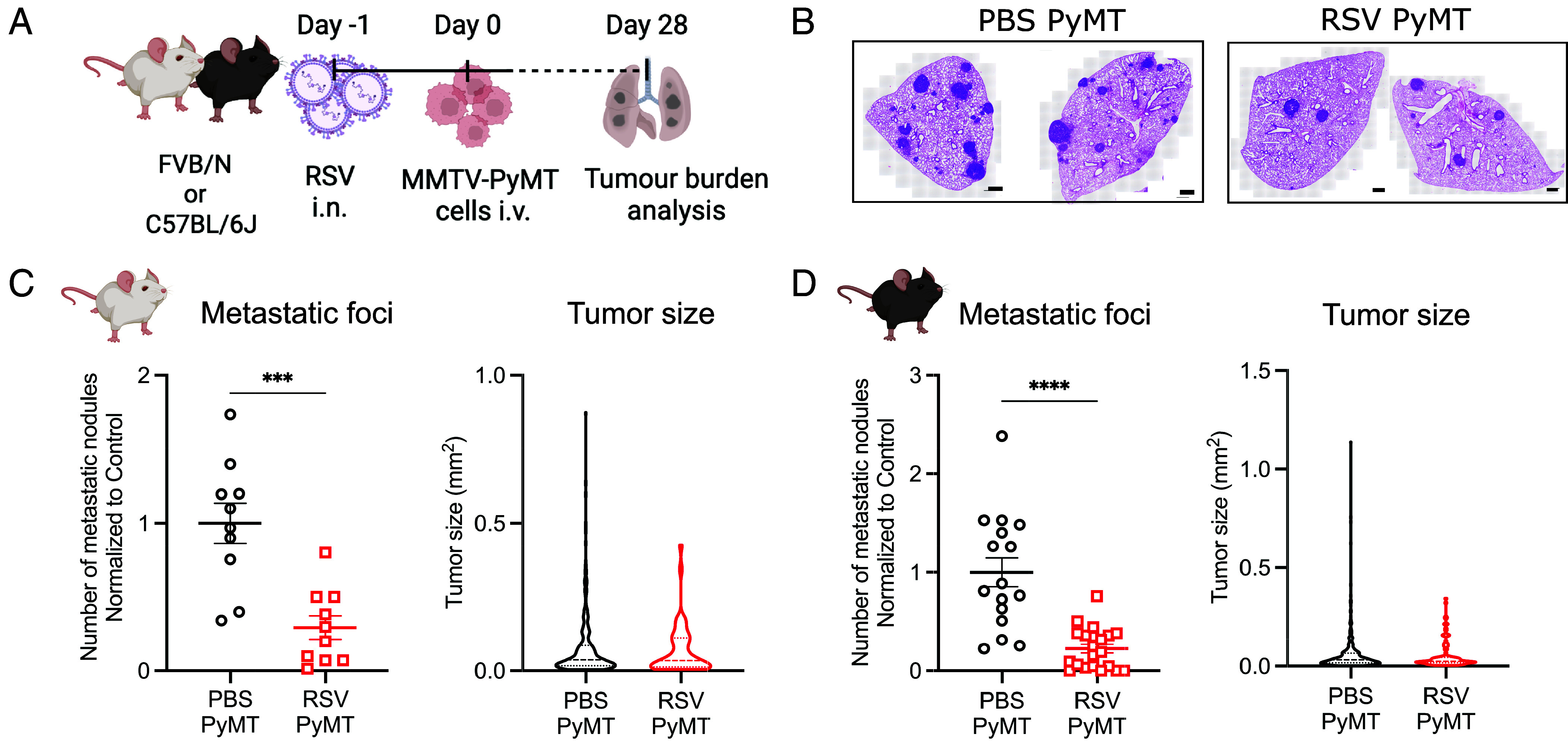
RSV infection impairs the development of metastases in the lungs. (*A*) Experimental setup. FVB/N or C57BL/6J mice were intranasally (i.n.) infected with RSV (RSV PyMT) or treated with PBS (PBS PyMT). A day later, 3 × 10^5^ MMTV-PyMT cells were intravenously (i.v.) injected. Tumor burden was analyzed 28 d later by assessing three levels at least 150 μm apart of all lobes by H&E staining. (*B*) Representative H&E-stained sections of PBS or RSV exposed lungs of FVB/N mice 28 d after PyMT i.v. injection. (Scale bar, 500 µm.) Total number of metastatic nodules in lungs from (*C*) FVB/N mice and (*D*) C57BL/6J mice were normalized to the average of the uninfected (PBS) group in each independent experiment. Tumor size shows the size of all tumors detected in all mice from each group. Data for FVB/N are pooled from two independent experiments presented as the mean ± SEM of 10 mice/group. Data for C57BL/6J are pooled from three independent experiments presented as the mean ± SEM of 16 mice for mock infected and 23 mice for the RSV infected group. Student’s *t* test was performed. Only statistically significant differences are shown; ***P < 0.001; ****P < 0.0001.

Direct injection of cancer cells into the circulation of mice is a well-established model for experimental metastasis, allowing for synchronized lung seeding and subsequent growth. To investigate if respiratory virus infection impacts breast cancer lung metastasis, we studied the ability of intravenously (i.v.) inoculated breast cancer cells (MMTV-PyMT cells) to seed and grow in the lungs of RSV-infected mice. Primary MMTV-PyMT cells were injected into FVB/N or C57BL/6J mice 24 h after intranasal (i.n.) infection with RSV (Fig. 1*A*). Tumor burden was assessed 28 d after cell administration by histological analysis and macroscopic nodule quantification. Interestingly, FVB/N and C57BL/6J mice displayed lower number of metastatic nodules in the lungs when they were previously infected with RSV (Fig. 1 *B–D* and *SI Appendix*, Fig. S1). However, the metastatic nodules that did develop in RSV infected lungs showed a similar size distribution to those in control mock-infected (Phosphate-Buffered Saline (PBS) instillation) mice (Fig. 1 *B–D*, *Left* panels). Similar results were found in BALB/c mice using 4T1 breast cancer cells (*SI Appendix*, Fig. S2). These data suggest that RSV infection inhibits metastatic lung colonization but that, once this occurs, tumor cells can grow unimpeded.

### The Presence of Metastatic Cells in the Lung Does Not Influence the Overall Response to RSV Infection.

**Fig. 2.**
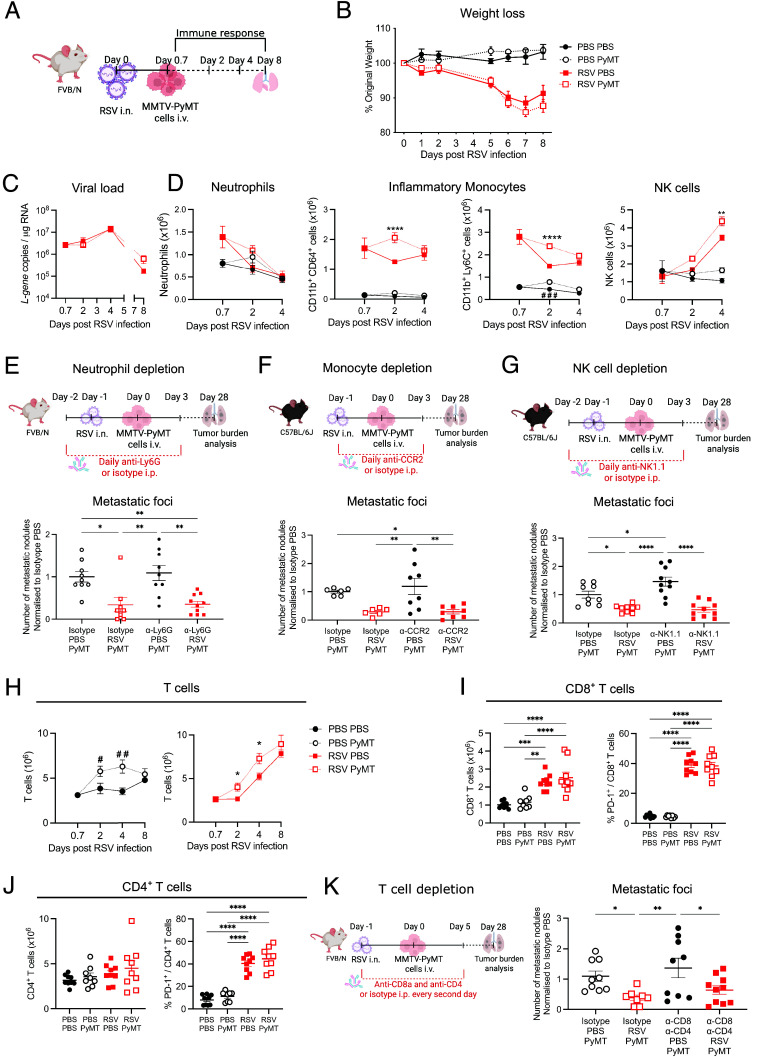
The role of RSV-induced immune responses in the antimetastatic effect observed following infection. (*A*) FVB/N mice were intranasally (i.n.) infected with RSV or treated with PBS. A day later, 3 × 10^5^ MMTV-PyMT cells were intravenously (i.v.) injected (PBS PyMT and RSV PyMT). (*B*) Disease severity was assessed by weight loss plotted as percentage of original weight. (*C*) Viral load was quantified by RT-qPCR at different time points after infection from RNA isolated from lung tissue. (*D*) Numbers of neutrophils, inflammatory monocytes (gated as CD11b^hi^ CD64^hi^ or CD11b^hi^ Ly6C^+^), and NK cells present in the lungs at different times during infection were analyzed by flow cytometry. (*E*) FVB/N mice were depleted of neutrophils by intraperitoneal (i.p.) treatment with 150 µg of anti-Ly6G or isotype control every second day starting a day prior to RSV infection up to Day 4 p.i. (*F*) C57BL/6J mice were depleted of monocytes by treating daily with 20 µg anti-CCR2 or isotype i.p. from the day of RSV infection until Day 5 p.i. (*G*) To deplete lung NK cells, 100 µg of anti-NK1.1 was administered i.n. a day before infection. Depletion was maintained until Day 4 p.i. by daily injection of 150 µg of anti-NK1.1. (*E*–*G*) MMTV-PyMT cells were injected i.v. a day after RSV infection as previously described. Metastatic burden was evaluated 28 d after tumor cell injection. (*H*) Analysis of the T cell response in lungs from FVB/N mice mock-infected (PBS) or infected i.n. with RSV and injected i.v. with MMTV-PyMT cells. Number of T cells (CD3^+^) recovered from lungs and detected by flow cytometry. Total (*I*) CD8^+^ T cells and (*J*) CD4^+^ T cells as well as activated T cells measured as PD1^+^ T cells were quantified in lungs Day 8 p.i. (*K*) CD4^+^ and CD8^+^ T cells were depleted by i.p. administration of anti-CD4 and anti-CD8 or isotype control every second day starting the day of infection until Day 6 p.i. MMTV-PyMT cells were injected i.v. a day after RSV infection. Tumor burden was assessed by H&E quantification 28 d after tumor cell administration. Weight loss data (*B*) are pooled from three independent experiments and shown as mean ± SEM of n = 13 for both infected groups and n = 12 for the MMTV-PyMT mock-infected group. Data for the PBS group are pooled from two experiments and shown as mean ± SEM of n = 8. A two-way ANOVA, mixed-effect analysis was performed to compare weight loss after infection followed by Tukey’s post hoc test. (*C*, *D*, and *H*–*J*) One-way ANOVA was performed to compare the PBS and PyMT mice (#) and the RSV and RSV PyMT mice (*) at each time point. All data are pooled from two experiments. (*E*–*G*, and *K*) One-way ANOVA was performed to compare all groups (*). For n numbers please refer to *SI Appendix*, Figs. S3, S5, and S6. Only statistically significant differences are shown; *P < 0.05, **P < 0.01, ***P < 0.001, ****P < 0.0001; #*P* < 0.05, *P* < 0.01, ### < 0.001.

Using FVB/N mice, we also assessed whether the presence of lung metastases influences the course of RSV infection (Fig. 2*A*). FVB/N mice developed disease during RSV infection (Fig. 2*B*), with increased weight loss compared to that which we have previously reported in C57BL/6J mice (21, 27). Interestingly, administration of MMTV-PyMT cells a day after infection did not alter subsequent weight loss (Fig. 2*B*) or viral load (Fig. 2*C*). The total number of cells, mostly CD45^+^, recovered from the lungs and the bronchoalveolar lavage (BAL) increased at Days 4 and 8 post infection (p.i.), respectively (*SI Appendix*, Fig. S3 *A–C*). Notably, increased numbers of lung cells were detected at 18 h and 3 d post MMTV-PyMT cell administration (d2 and d4 p.i.) irrespective of RSV infection (*SI Appendix*, Fig. S3*A*).

To further characterize the immune response induced by RSV in the presence of tumor cells, different cell types were quantified by flow cytometry (gating strategy *SI Appendix*, Fig. S3*D*) in the lungs (Fig. 2*D* and *SI Appendix*, Fig. S3*E*) and in the BAL (*SI Appendix*, Fig. S3*F*) at different time points. RSV infection did not alter the number of alveolar macrophages (AMs; *SI Appendix*, Fig. S3 *E* and *F*). However, the infection resulted in recruitment of neutrophils, with higher numbers detected 18 h p.i. (0.7 d) [Fig. 2 *D*, *Left* panel, (28)]. Similarly, inflammatory monocytes (gated as Ly6G^−^ SiglecF^−^ CD11b^+^ CD64^+^ cells or Ly6G^−^ SiglecF^−^ CD11b^+^ Ly6C^+^ cells) were detected as early as 18 h, with higher numbers in the PyMT-bearing mice at Day 2 p.i. (Fig. 2 *D*, *Middle* panel). Furthermore, an increase in the abundance of NK cells, peaking at 4 d p.i., with higher numbers in the RSV-infected mice that also received PyMT cells were detected (Fig. 2 *D*, *Right* panel).

We next examined immune mediator expression following RSV infection in the presence of tumor cells. IL-6 and IFN-α levels in BAL fluid (*SI Appendix*, Fig. S4*A*) and lung mRNA levels (*SI Appendix*, Fig. S4*B*) were similar across infected groups. Likewise, mRNA levels of *Ifnb*, *Ifnl*, *Il1b*, *Ccl2*, and *Cxcl1* (*SI Appendix*, Fig. S4*C*) showed no differences regardless of tumor cell injection. Interferon-stimulated genes (ISGs) (*Pkr*, *Viperin*, *Cxcl10*, *Mx1*, *Oas1*) were similarly induced postinfection (*SI Appendix*, Fig. S4*D*). These findings suggest that RSV-driven innate immune responses are largely unaffected by tumor cells seeding the lungs, aside from a transient increase in inflammatory monocytes, NK cells, and T cells when infection proceeds concomitantly with the presence of tumor cells.

### Neutrophils, Monocytes, or NK Cells Are Not Essential to Impair Tumor Cell Metastases During RSV Infection.

Type I IFNs are produced by AMs upon detection of RSV (21). Interestingly, type I IFNs can induce an antitumor response by activating innate immune cells, including neutrophils, monocytes, and NK cells (22, 26, 29–32). To assess a potential role of neutrophils in the antitumoral response, we used anti-Ly6G antibody-mediated neutrophil depletion during RSV infection (Fig. 2 *E*, *Top* panel and *SI Appendix*, Fig. S5*A*). Interestingly, no differences in numbers of metastatic foci were detected between infected mice with or without neutrophils (Fig. 2 *E*, *Bottom* panel). Neutrophil depletion also had no impact on RSV-induced disease severity (*SI Appendix*, Fig. S5*B*). We have previously shown that mice lacking MyD88 and TRIF adaptor proteins are unable to recruit neutrophils to the lungs during RSV infection (28). MyD88/TRIF deficient mice were infected with RSV and inoculated with tumor cells i.v. a day later. Similar to neutrophil depletion, RSV-infected *Myd88/Trif^-/−^* mice and wildtype mice displayed a similar decrease in number, but not size, of metastatic nodules compared to noninfected controls (*SI Appendix*, Fig. S5*C*). Together, these data suggest that RSV-induced recruitment of neutrophils to the lungs is not necessary for reducing lung metastatic colonization.

We next studied the role of monocytes. Monocytopenia was achieved using anti-CCR2 antibodies (33) from the day of infection until Day 5 p.i. (Fig. 2 *F*, *Top* panel and *SI Appendix*, Fig. S5*D*). Disease severity caused by RSV infection was not altered in the absence of monocytes (*SI Appendix*, Fig. S5*E*). Lack of monocytes did not impact the number of metastatic foci as this was equally reduced in isotype-matched control and anti-CCR2 treated mice after RSV infection (Fig. 2 *F*, *Bottom* panel).

RSV infection also causes an increase in the number of NK cells in the lungs, which is further potentiated by the presence of cancer cells (Fig. 2 *D*, *Right* panel). We therefore depleted NK cells using anti-NK1.1 antibodies before and during RSV infection (Fig. 2 *G*, *Top* panel and *SI Appendix*, Fig. S5*F*). This did not impact weight loss caused by the infection (*SI Appendix*, Fig. S5*G*). As expected, NK cell depletion in mock-infected mice led to an increase in lung metastases but, strikingly, did not curtail the inhibitory effect of the infection on metastatic establishment (Fig. 2 *G*, *Bottom* panel). These results indicate that NK cells are not necessary for the reduction in lung metastatic initiation caused by RSV infection. Altogether, our results show that despite the RSV-dependent increase in neutrophils, monocytes, and NK cells, each of those immune cells alone does not explain the virus-induced antitumoral response.

### The T Cell Response During RSV Infection Does Not Play an Essential Role in Decreasing the Metastatic Burden.

The presence of cancer cells transiently increased the number of T cells at Days 2 and 4 p.i. (Fig. 2 *H*, *Left* panel), while RSV infection induced recruitment of T cells detectable as early as Day 4 p.i. However, increased numbers of pulmonary T cells are detected at Day 2 in the presence of cancer cells in infected lungs (Fig. 2 *H*, *Right* panel). At Day 8 post RSV infection more CD8^+^ and CD4^+^ T cells were detected in lung and BAL, yet numbers were unaltered by presence of PyMT cells (Fig. 2 *l* and *J*, *Left* panels and *SI Appendix*, Fig. S6*A*). Activation of T cells, as determined by expression of PD1 and CD69, was also similar in all infected mice irrespective of inoculation with tumor cells (Fig. 2 *I* and *J*, *Right* panels and *SI Appendix*, Fig. S6 *B* and *C*). Further, levels of IFN-γ and Granzyme B in the BAL were similar across infected groups (*SI Appendix*, Fig. S6*D*). Overall, these results suggest that the T cell response induced by RSV infection is not markedly affected by the presence of MMTV-PyMT cells in the lungs.

As T cells were elevated in infected lungs, we assessed their potential role in the antitumoral effect induced by RSV infection. CD8^+^ and CD4^+^ T cell depletion (Fig. 2 *K*, *Left* panel and *SI Appendix*, Fig. S6*E*), as previously shown (34), ameliorates weight loss at Day 5 to 7 p.i. (*SI Appendix*, Fig. S6*F*). However, regardless of the presence of T cells, RSV infection still led to a lower number of metastatic nodules compared to uninfected mice (Fig. 2 *K*, *Right* panel). Thus, T cells are not necessary for the reduced metastatic burden induced by RSV infection.

### Type I IFNs Recapitulate the Antitumoral Effect Observed After RSV Infection.

Type I IFNs are key antiviral cytokines released early upon RSV infection and responsible for inhibiting viral replication and orchestrating the innate immune response by signaling through the IFNα/β receptor (IFNAR1) (16). We therefore analyzed how IFN-α changes the lung environment shortly after exposure (*SI Appendix*, Fig. S7*A*). Administration of IFN-α i.n. to C57BL/6J or FVB/N mice resulted in a transient inflammatory response characterized by activation of resident neutrophils (determined as CD64^+^ neutrophils) and recruitment of inflammatory monocytes [*SI Appendix*, Fig. S7 *B–D*, (16)]. Expression of several chemokines such as *Ccl2* and *Cxcl1* and cytokines including *ll1b and Il6* and ISGs was also increased at this time point (*SI Appendix*, Fig. S7 *E* and *F*). Thus, inhalation of IFN-α induces changes in the lung environment that are still detectable at 18 h postexposure. To note, the presence of MMTV-PyMT cells did not impact the overall lung response to IFN-α administration except for increased neutrophil activation (*SI Appendix*, Fig. S8).

**Fig. 3.**
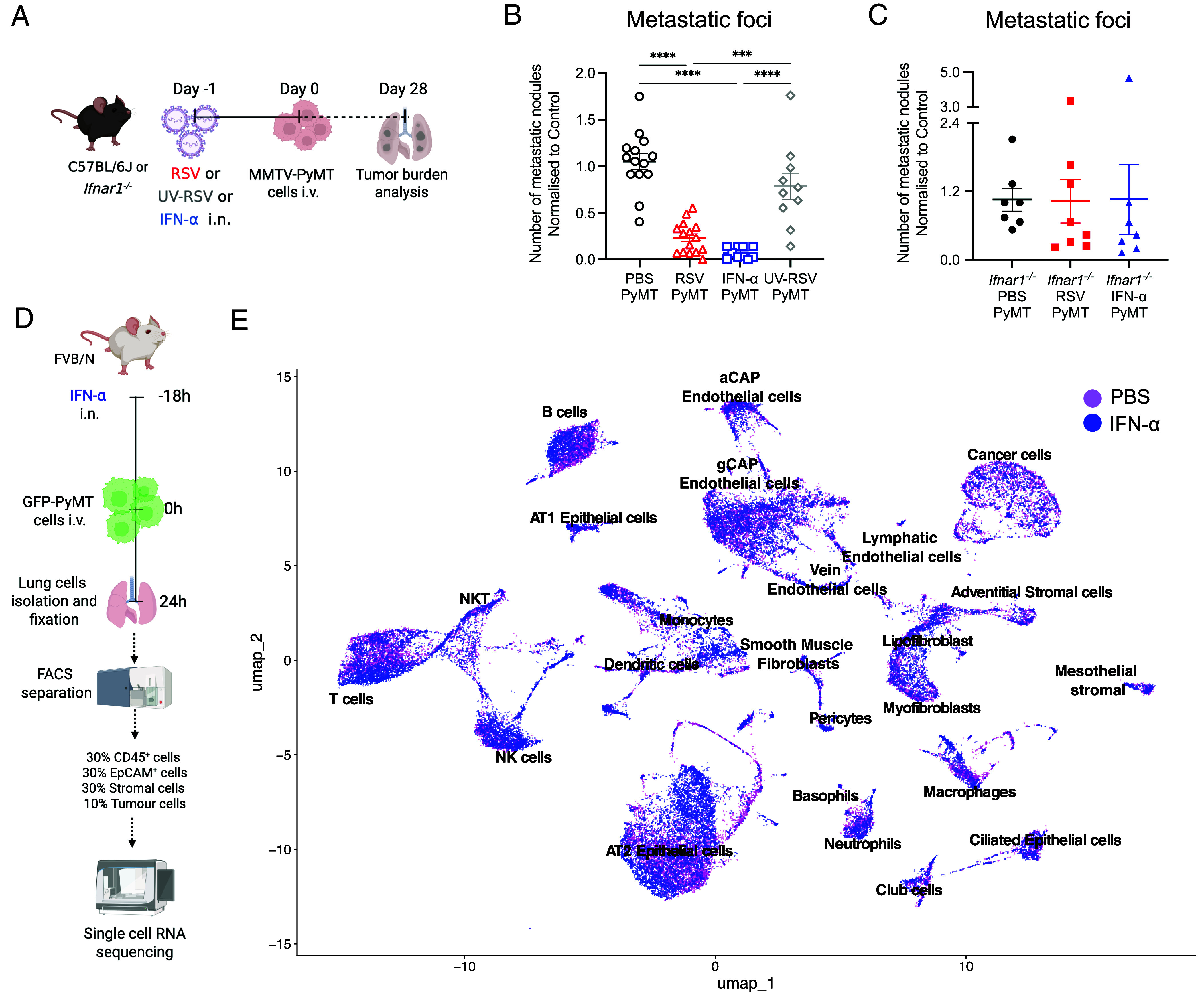
Reduced numbers of metastatic nodules in the lung following RSV infection are due to type I IFN receptor signaling. (*A*) Experimental setup. C57BL/6J or *Ifnar1^−/−^* mice were intranasally mock infected (PBS PyMT), infected with RSV (RSV PyMT), exposed to two doses of 500 ng IFN-α (18 h apart, with the second dose at least 4 h before tumor cell injection; IFN-α PyMT) or exposed to UV-inactivated RSV (UV-RSV PyMT). MMTV-PyMT cells were injected i.v. a day later. Number of metastatic nodules was determined by H&E staining of three levels of each lobe, 28 d after tumor cell injection in (*B*) C57BL/6J mice and (*C*) *Ifnar1^−/−^* mice. Total number of metastatic tumors was normalized to the average number of tumors in the uninfected group in each independent experiment. Data are presented as the mean ± SEM pooled from two independent experiments, for C57BL/6J mice n = 14 for PBS PyMT, n = 15 for RSV PyMT, n = 10 for IFN-α PyMT and UV-RSV PyMT. For *Ifnar1^−/−^* mice n = 7 to 8 for PBS PyMT, IFN-α PyMT and RSV PyMT. One-way ANOVA test followed by Tukey’s post hoc test was performed to compare all groups. Only statistically significant differences are shown; ***P < 0.001; ****P < 0.0001. (*D*) Experimental setup. FVB/N mice were intranasally exposed to PBS or 1 µg IFN-α IFN-α, GFP-expressing MMTV-PyMT cells were injected i.v. 18 h later. Lungs were collected after 24 h, and single cells were obtained, stained, fixed and sorted before scRNA sequencing and analysis. (*E*) UMAP plot of scRNAseq data from lungs of PBS and IFN-α exposed PyMT-injected mice.

To determine if type I IFNs phenocopy the antitumoral effect of RSV infection, we administered two doses of recombinant IFN-α intranasally to C57BL/6J mice, 24 and 18 h prior to cancer cells injection and assessed tumor burden 28 d later (Fig. 3*A*). Remarkably, compared to the control group, IFN-α exposure resulted in reduced number of metastatic nodules in the lung, to a similar extent as RSV infection (Fig. 3*B*). Exposure to UV-inactivated RSV, which does not induce expression of type I IFNs (35), did not reduce the metastatic burden (Fig. 3*B*). In line with these results, RSV infection in mice lacking the type I IFN receptor -IFNAR1 (*Ifnar1^−/−^* mice; Fig. 3*A*) did not have an impact on the metastatic burden (Fig. 3*C*). To further assess if IFN-α impacts tumor cells directly, *Ifnar1^−/−^* mice were treated with IFN-α to create a setting in which only the tumor cells can respond to the cytokine. The number of metastatic nodules in *Ifnar1^−/−^* mice was not altered after exposure to IFN-α (Fig. 3*C*). Overall, these data suggest that IFN-α induces changes in the lung environment rather than acting directly on the tumor cells to reduce metastatic initiation.

We next studied how IFN-α influences the interaction of lung cells with the metastatic cells by performing single cell RNA sequencing. Mice were exposed to IFN-α or PBS 18 h prior to tumor cell injection and the lungs were collected 24 h later (Fig. 3*D*). To balance representation of different lung cell populations, cancer, epithelial, immune, and mesenchymal cells were sorted and processed for single cell RNA analysis at a ratio of 1:3:3:3, respectively (Fig. 3*D*). The different cell populations, from mock or IFN-α treated mice, were clustered (Fig. 3*E*) and changes in the different cellular compartment following exposure to IFN-α were revealed by UMAP (*SI Appendix*, Fig. S9 *A–D*). In line with our previous results, when studying immune cells via CellChat analysis it was confirmed that after IFN-α exposure only minor alterations of the interaction between immune cells and cancer cells were apparent (*SI Appendix*, Fig. S9 *A* and *E*).

### Lung Epithelial Cells from RSV Infected or IFN-α Exposed Mice are Less Supportive of MMTV-PyMT Cell Growth.

**Fig. 4.**
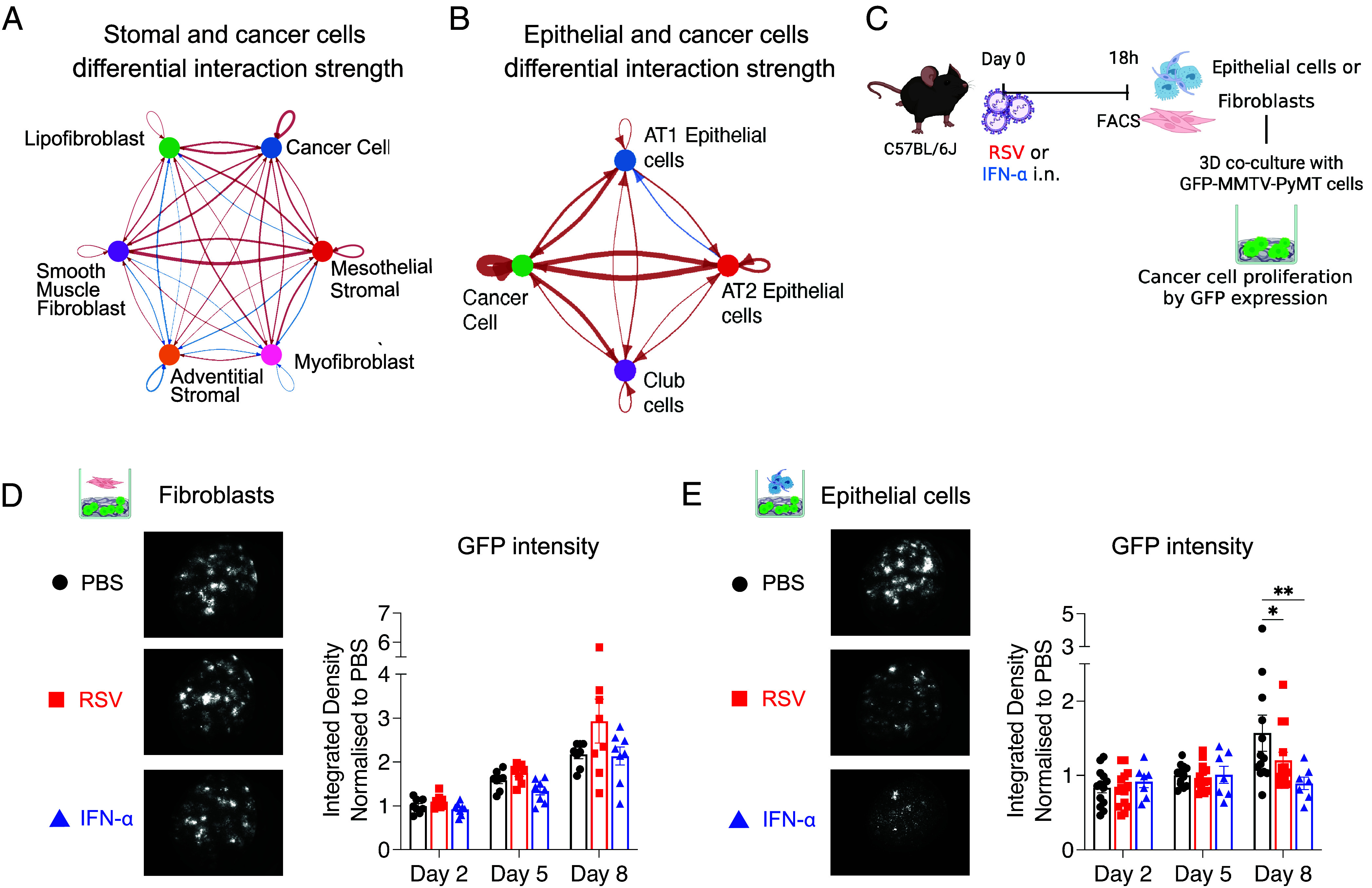
Lung epithelial cells from RSV infected mice or mice exposed to IFN-α are less supportive of tumor cell proliferation. CellChat analysis was used to analyze scRNAseq obtained as described in Fig. 3*D* and Circle plot of Differential Interaction Score of Cancer Cells with (*A*) stromal cell or (*B*) epithelial cell compartments was created based on Secreted ligands and Receptor interactions. (*C*) Experimental setup. IFN-α, PBS or RSV was intranasally administered to C57BL/6J. 18 h later, fibroblasts and epithelial cells were sorted via FACS. Each cell type was cocultured with GFP-expressing MMTV-PyMT cells in a collagen-solution-coated Alvatex Scaffold 96-well plate. Tumor cell proliferation was monitored daily for 8 d using the SteREO LumarV12 stereomicroscope. Images of (*D*) fibroblasts or (*E*) epithelial cells cultures are representative of the cocultures at Day 8. All data points were normalized to the mean integrated density of the PBS group at Day 2. Data are shown as mean ± SEM. Data from fibroblast cocultures are pooled from two independent experiments with eight wells per condition. Data from cocultures with epithelial cells are pooled from two-three independent experiments, n = 13 to 14 wells for PBS and RSV and n = 7 wells for IFN-α. Statistical differences were assessed by two-way ANOVA with Tukey’s multiple comparisons. Only statistically significant differences are shown; *P < 0.05; **P < 0.01.

Fibroblasts are a dominant cell type in the tumor niche but only minimal changes in the interaction between mesenchymal and cancer cells were apparent upon IFN-α exposure (Fig. 4*A* and *SI Appendix*, Fig. S9*B*). In contrast, IFN-α had a striking effect on the interactions between lung epithelial cells and cancer cells (Fig. 4*B* and *SI Appendix*, Fig. S9*C*). We therefore evaluated if exposure to RSV or IFN-α affects the interactions between epithelial cells and tumor cells to impact early cancer cell growth. Mice were given either RSV, IFN-α or PBS i.n. and fibroblasts (CD45^−^CD31^−^Epcam^−^) and epithelial cells (CD45^−^CD31^−^Epcam^+^) were isolated by Fluorescence-Activated Cell Sorting (FACS) after 18 h (gating strategy *SI Appendix*, Fig. S9*F*). An Alvetex™ scaffold coculture system that mimics the 3D tissue environment was used to culture GFP-expressing MMTV-PyMT cells in the presence of either fibroblasts or epithelial cells (Fig. 4*C*). Tumor cell growth was subsequently followed over time by the intensity of GFP signal. Cocultures with fibroblasts from PBS-exposed mice induced tumor cell proliferation, detectable as early as Day 5, which was not altered in the presence of fibroblasts from mice infected with RSV or exposed to IFN-α (Fig. 4*D*). In contrast, coculture of tumor cells with epithelial cells from mock-treated mice showed significantly enhanced cancer cell proliferation at Day 8. Interestingly, epithelial cells isolated from RSV or IFN-α exposed lungs failed to support cancer cell proliferation to the same levels seen in the control group (Fig. 4*E*). These results demonstrate that epithelial cells from lungs of RSV-infected or type I IFNs exposed mice are less able to support tumor cell proliferation.

### MMTV-PyMT Cell Seeding, and Early Tumor Cell Growth Is Blunted after RSV Infection or IFN-α Administration.

**Fig. 5.**
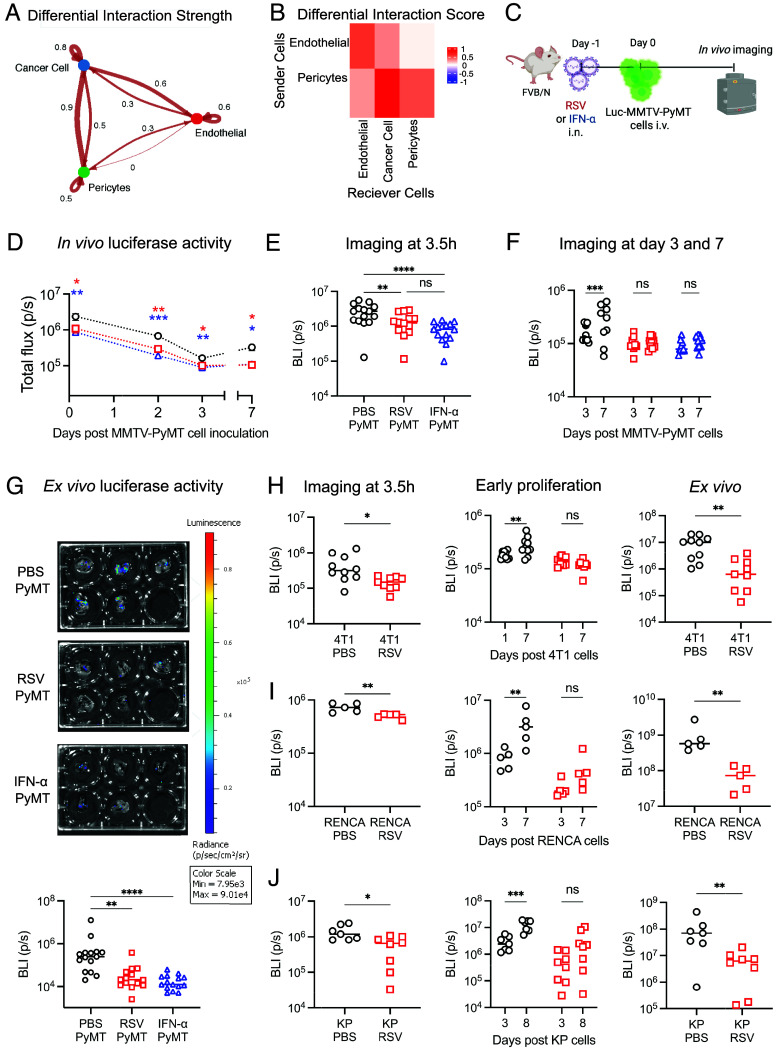
RSV infection inhibits tumor cell seeding in the lung and early cancer cell growth in a type I IFN dependent manner. (*A*) CellChat analysis was performed on the scRNAseq dataset to create Circle plot of Differential Interaction Score of Cancer Cells with vascular cell components based on Secreted ligands and Receptor interactions. (*B*) Heatmap of Differential Interaction Score showing endothelial and pericytes as sender cells and endothelial, cancer cells and pericytes as receivers. (*C*) FVB/N mice were intranasally treated with PBS (PBS PyMT), one dose of 1 µg of IFN-α (IFN-α PyMT) or infected with RSV (RSV PyMT). A day later luciferase expressing MMTV-PyMT cells were injected i.v. (*D*) At different time points after cell injection, luminescence signal (BLI, photons/second) was quantified using Living Image Software by selecting a region of interest (ROI) of a set size positioned over the thorax of each animal. (*E*) In vivo quantification of luciferase activity 3.5 h after MMTV-PyMT cell injection. (*F*) Comparison of in vivo luciferase activity at Day 3 and 7 for each experimental group. (*G*) Ex vivo imaging was performed at Day 28 to determine tumor burden, representative images from the different group are shown as well as the quantification of the luciferase activity pooled from three independent experiments, n = 15 per group. Seeding and early growth of tumor cells after RSV infection was also assessed using (*H*) 3 × 10^5^ luciferase expressing-4T1 cells in BALB/c mice, (*I*) 4 × 10^5^ luciferase expressing-RENCA cells in BALB/c mice or (*J*) 3 × 10^5^ luciferase expressing-KP1233 cells in C57BL/6J mice. (*D*–*G*) Data are shown as mean ± SEM, pooled from three independent experiments for Day 0, Day 2, and Day 28 (n = 15) and two independent experiments for Day 3 and 7 (n = 10). (*H*) Data are pooled from two independent experiments, n = 9 to 10. (*I*) Data are shown from one experiment, n = 5. (*J*) Data are pooled from two independent experiments, n = 7 to 8. Statistical significance was determined by (*D*) Two-way ANOVA with Tukey’s multiple comparisons, (*E* and *F*) One-way ANOVA test followed by Tukey’s post hoc test was performed to compare all groups and (*G*) Kruskal–Wallis test with Dunn’s multiple comparisons statistical analysis against the PBS group (for the ex vivo readout). (*H*–*J*) To compare PBS and RSV groups only, Student’s *t* test was performed. Only statistically significant differences are shown; *P < 0.05; **P < 0.01; ***P < 0.005; ****P < 0.001, ns = not significant.

To further understand the effect of type I IFNs on the homing and seeding of tumor cells, we assessed interactions between the lung vasculature and cancer cells, which enter the tissue via the circulation. While no changes were detected in the interaction between endothelial cells and pericytes, we could observe an increase in their interactions with cancer cells upon IFN-α exposure (Fig. 5 *A* and *B* and *SI Appendix*, Fig. S9*D*). To test whether this could influence the ability of cancer cells to seed the lung, we followed in vivo tumor cell extravasation by injecting luciferase-expressing MMTV-PyMT cells into mice after exposure to RSV or IFN-α (Fig. 5*C*). Bioluminescence was monitored at different time points after cell injection. Notably, RSV infection, as well as IFN-α administration, resulted in a reduced luciferase signal in the lung as early as 3.5 h post injection, indicating a reduced ability of circulating cancer cells to seed the lung (Fig. 5 *D* and *E*). This was confirmed by flow cytometry, using GFP-expressing MMTV-PyMT cells (*SI Appendix*, Fig. S10 *A–C*). Therefore, the initial seeding of lungs by circulating cancer cells is restricted by RSV infection, as well as IFN-α administration, likely reflecting the impact of the cytokines on the lung endothelium. Furthermore, the expected reduction in luciferase signal detected at 2- and 3-d post injection was very similar in all three experimental groups, suggesting that the extravasation of the metastatic cells into the lung parenchyma was not affected by exposure to RSV or IFN-α (Fig. 5*D*).

In lungs from control mice, we observed an increase in the luciferase signal from Day 3 to Day 7, indicative of the ability of MMTV-PyMT cells to initiate early growth post extravasation (Fig. 5*F*). Interestingly, this increase was not seen in lungs from mice infected with RSV or exposed to IFN-α. Consistent with this finding, GSEA analysis indicated that cancer cells in IFN-α pre-exposed lungs display a reduction in pathways linked to cell cycle and DNA replication, (*SI Appendix*, Fig. S10*D*). The combination of a reduction in extravasation and a restriction in early growth resulted in an overall reduction in bioluminescence ex vivo at the Day 28 endpoint, which was confirmed by histological quantification to reflect lower tumor burden (Fig. 5*G* and *SI Appendix*, Fig. S10*E*). Finally, we confirmed that this was not restricted to the MMTV-PyMT model by showing that RSV infection impaired seeding and early tumor growth in experimental lung metastasis models using a breast cancer cell line (4T1), a renal cancer cell line (RENCA) and a lung cancer cell line (KP1233 cells) (Fig. 5 *H–J*). We conclude that RSV infection or IFN-α exposure renders the lung environment more hostile for cancer cell seeding and early metastatic growth.

### Galectin-9 has a Direct Effect on Cancer Cells Leading to a Reduced Cell Seeding in the Lungs.

**Fig. 6.**
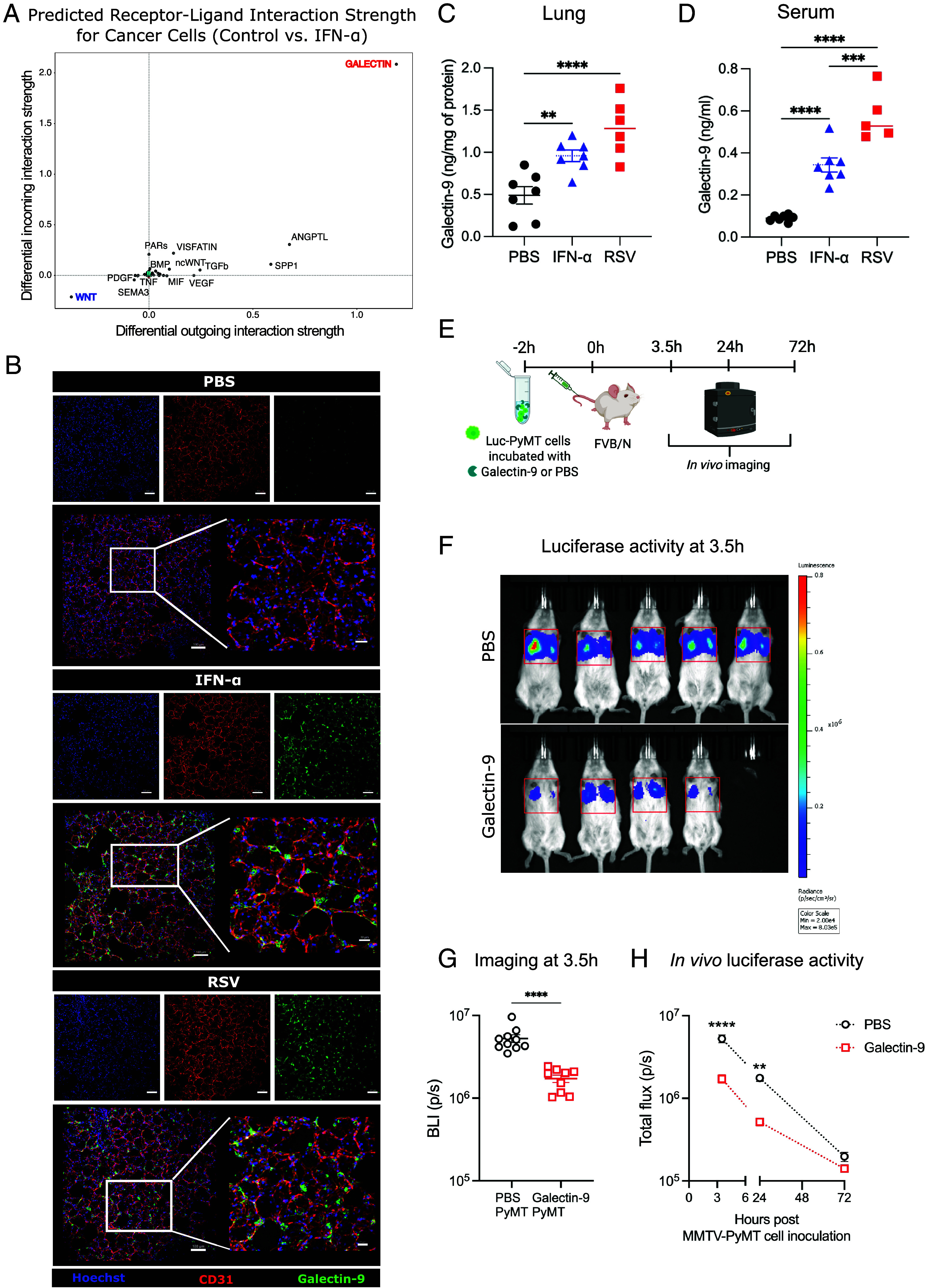
Galectin-9 affects MMTV-PyMT cell seeding into the lung. CellChat analysis was performed from scRNA Sequencing data shown in Fig. 3*D*. (*A*) Plot of differential outgoing and incoming interaction strength for receptor–ligand interaction pathways that affect the Cancer Cell cluster, comparing cells from MMTV-PyMT-GFP injected, PBS vs. IFN-α treated mice. (*B*) FVB/N mice were intranasally exposed to RSV or IFN-α. After 18 h, lungs were harvested. The left lung lobe was inflated with agarose and 300 μm sections were stained for CD31, Galectin-9, and Hoechst. (Scale bars, 100 μm and 30 μm in the *Inset*.) Images shown are representative from three mice per condition. (*C*) The top right lung lobe was homogenized and used to quantify levels of Galectin-9 by ELISA in lung homogenates. (*D*) Serum from these mice was used to quantify circulating Galectin-9 by ELISA. (*E*) Luciferase-expressing MMTV-PyMT were exposed to recombinant Galectin-9 for 2 h and then injected i.v. in FVB/N mice, (*F*) IVIS imaging of animals at 3.5 h post i.v. injection. Red boxes show the area for the calculated ROI. (*G*) Quantification of bioluminescence ROIs at 3.5 h post injection of MMTV-PyMT-luciferase cells. (*H*) Time course of bioluminescence signal over 72 h from injection. (*C* and *D*) All data are shown as mean ± SEM of n = 6 to 7 mice per group from two pooled experiments. One-way ANOVA test followed by Tukey’s post hoc test was performed to compare all groups. (*G* and *H*) All data are shown as mean ± SEM of n = 9 to 10 mice per group pooled from two independent experiments. Statistical differences were assessed by Student’s *t* test with Welch correction; **P < 0.01, ****P < 0.001.

CellChat analysis of ligands likely to be acting on cancer cells infused into IFN-α treated mice revealed Galectins as the strongest hit (Fig. 6*A*). This led us to explore whether Galectins might be downstream of type I IFNs in restriction of cancer metastases. In particular, Galectin-9 is a well-established ISG (36). Interestingly, we found that Galectin-9 mRNA was strongly upregulated in most cell types in the lungs upon intranasal delivery of IFN-α or infection with RSV (*SI Appendix*, Fig. S11*A*). This was also confirmed by immunohistochemistry (*SI Appendix*, Fig. S11*B*). For greater anatomical resolution, we carried out Galectin-9 staining in precision cut lung slices (PCLS) 18 h post RSV infection or IFN-α exposure. This analysis confirmed widespread staining throughout the lung parenchyma, often in or around endothelial cells of the alveolar structure (Fig. 6*B*), but not the larger airways (*SI Appendix*, Fig. S11*C*). Consistent with its wide distribution and abundance, Galectin-9 was also detected in lung homogenate (Fig. 6*C*) and, and in the airways (BAL; *SI Appendix*, Fig. S11*D*), as well as systemically in the serum (Fig. 6*D*).

Finally, we assessed the impact of Galectin-9 on cancer cell lung seeding by incubating luciferase-expressing MMTV-PyMT cells with Galectin-9 before i.v. injection (Fig. 6*E*). To note, Galectin-9 did not influence the viability of luciferase-expressing MMTV-PyMT cells (*SI Appendix*, Fig. S11*E*) nor alter the expression of genes such as E-Cadherin, N-Cadherin, Vimentin, or α-SMA, associated with epithelial–mesenchymal transition, drivers of metastatic potential (*SI Appendix*, Fig. S11*F*). However, mice given MMTV-PyMT-luciferase cells pretreated with Galectin-9 displayed lower luciferase activity in the lungs (Fig. 6 *F–H*). This was detected as early as 3.5 h after tumor cell administration (Fig. 6 *F–H*), consistent with restriction in metastatic seeding, thereby phenocopying the effects observed in mice pre-exposed to IFN-α or infected with RSV (Fig. 5 *C–F*). These results suggest that Galectin-9, induced by type I IFN receptor signaling, can impair cancer metastasis initiation and may be one of the mediators of IFN-dependent metastatic restriction.

## Discussion

In steady state, the lungs can serve as a niche propitious for cancer metastases (2). However, they are also exposed to respiratory viruses, which trigger acute inflammation and alter the tissue environment. The interplay between respiratory viral infections and the initiation and progression of lung metastasis remains unclear. Here, using several experimental cancer metastasis models, we show that early events following RSV infection reduce the seeding and lung colonization of cancer cells. In addition, we find that virus-induced type I IFNs are key mediators of this effect, in part via epithelial and endothelial cells and the induction of Galectin-9.

Viral infections induce early production of type I IFNs (17). During RSV infection, AMs are the first and main producers of type I IFNs (21). The type I IFN response is very transient and has already started to decline when cells such as monocytes and NK cells are recruited to the lungs (21). The type I IFN receptor, IFNAR1, is expressed on all nucleated cells, therefore influencing all lung cell types. Moreover, signaling through the IFNAR1 potentiates IFN production through a positive feedback loop and induces expression of ISGs, which can limit viral replication and cause activation of neutrophils, inflammatory monocytes, NK cells, dendritic cells, and macrophages (16, 21, 28). We found that intranasal delivery of IFN-α could mimic the anti-metastatic effect induced by RSV infection in the lung, suggesting that IFNAR1 signaling during RSV infection creates an unfavorable environment for cell metastases. Interestingly, in several other metastatic tumor models (melanoma; B16, K1735m, and DX3, fibrosarcoma; UV2237) it has been reported that prophylactic i.p. administration of human hybrid IFN-α also reduces the number of lung metastases, further supporting a role for type I IFNs in decreasing the seeding and colonization of metastatic cells (37–39). Although type I IFNs can induce senescence or apoptosis of tumor cells (23), we show that intranasal administration of IFN-α to *Ifnar1*^−/−^ mice (where only the MMTV-PyMT cells express the type I IFN receptor) had no impact on metastatic burden. Thus, the effect triggered by type I IFNs in our model is not due to a direct effect on tumor cells.

We assessed the possible antitumoral role of immune cells that are recruited and activated by RSV infection. Interestingly, blockade of neutrophil recruitment or depletion of neutrophils, NK cells, monocytes, or T cells did not abrogate the anti-metastatic effect of RSV infection. While these experiments fail to demonstrate the necessity of any given immune cell type, multiple immune cell types working together, in a redundant manner, could still contribute to an anti-metastatic effect of RSV infection. Nevertheless, our data argue for a dominant effect of type I IFNs in impacting nonimmune lung cells to mediate restriction of metastatic cell seeding and growth.

Infiltrating cancer cells interact with cells in the lung parenchyma, which supports the initiation of metastatic growth (40–42). For example, lung epithelial cells play an essential role in supporting tumor cell growth (43–46) and they are also host cells for RSV replication (18). During respiratory viral infections, communication between lung epithelial cells, stromal cells, and immune cells is critical for an effective viral clearance without excessive inflammation (47). CellChat analysis showed that epithelial cells and cancer cells interact differently following IFN-α exposure compared to mock treated mice. Moreover, we show that RSV- or IFN-α-primed lung epithelial cells are less supportive of MMTV-PyMT cell growth. The key changes in epithelial cells induced by RSV infection or type I IFNs remain to be determined. This could be directly by signaling through IFNAR1 in epithelial cells or indirectly via type I IFNs activating other stromal cells, such as fibroblasts (48), which then act on epithelial cells.

Interestingly, analysis of our scRNAseq dataset suggested Galectins as strong candidates for proteins acting on cancer cells after infusion into mice treated with IFN-α. In parallel, robust expression of Galectin-9, an ISG associated with control of immunopathology during RSV infection of mice (49), was strongly upregulated in lung cell populations upon RSV infection or administration of IFN- α. Galectin-9 has been extensively studied in cancer as it can have dual roles in promoting and inhibiting tumor growth, immunomodulatory roles, and inducing immune evasion and promote neoplastic progression (50). In breast cancer, Galectin-9 has been suggested to have anti-metastatic potential by inducing tumor cell aggregation and reducing adhesion of breast cancer cells to the extracellular matrix (51–53). We confirmed that cancer cells treated with Galectin-9 display lower seeding of the lungs, suggesting that Galectin-9 could be one mediator of metastatic restriction after viral infection. In this regard, our finding of substantial levels of Galectin-9 in serum of mice infected with RSV or exposed to IFN-α is consistent with the possibility that cancer cells can be exposed to Galectin-9 in the bloodstream prior to arrival in the lung. Notably, Galectin-9 has also been detected in serum of COVID-19 patients, as well as in humans infected with influenza A virus, with a peak of expression during the acute phase of infection (54–56).

In sum, our data demonstrate that impaired metastatic cell homing, survival, and/or early proliferation in the lungs of RSV infected mice is likely mediated by rapid changes to the nonimmune compartment induced by type I IFNs. The IFNs can act, for example, by increasing Galectin-9 expression and/or changing interactions between epithelial/endothelial cells and cancer cells and impact seeding and early growth. Further human studies will be important to support our findings that respiratory viral infections can alter metastatic tumor progression in mice and assess how they can be potentially translated into clinical practice.

## Materials and Methods

### Mice.

C57BL/6J, FVB/N, and BALB/c mice were purchased from Charles River. *Ifnar1^−/−^* mice (on a C57BL/6 background) and *Myd88/Trif^−/−^* mice (obtained from S. Akira) (21) were bred in-house. Mouse mammary tumor virus-polyoma middle tumor-antigen (MMTV-PyMT) mice on an FVB/N or C57BL/6J background, FVB/N MMTV-PyMT actin-GFP and FVB/N MMTV-PyMT actin-luciferase were bred in-house (at The Francis Crick Institute). For all experiments age-matched (7 to 12 wk) female animals were used. All animal experiments were reviewed and approved by the Animal Welfare and Ethical Review Board within Imperial College London and The Francis Crick Institute and approved by the UK Home Office in accordance with the Animals (Scientific Procedures) Act 1986 and the ARRIVE guidelines.

### Infections and Treatments.

For intranasal administration, mice were lightly anesthetized with IsoFlo (isoflurane, Abbott Animal Health) and given 6 to 7 × 10^5^ focus-forming units RSV or UV-RSV in 100 μL. For IFN-α (Miltenyi Biotec) exposure, one dose of 1 µg or two doses of 500 ng 18 h apart in 100 μL were intranasally administered (16, 21). Antibody-mediated neutrophil, monocyte, NK cell, or T cell depletion was performed as detailed in *SI Appendix*, *Methods*.

### Experimental Lung Metastases.

Primary cells were isolated from MMTV–PyMT breast tumors as previously described (40). 0.3 × 10^6^ MMTV-PyMT or MMTV-PyMT-GFP 0.5 × 10^6^ cells were inoculated intravenously (i.v.) in 200 μL. For in vivo imaging, refer to the section below.

### Flow Cytometry.

Lung and BAL cells (obtained as described in *SI Appendix*, *Methods*) were stained in 50 μL with fixable live-dead Aqua dye (Invitrogen) and different fluorochrome-conjugated antibodies (*SI Appendix*, Table S1) for 25 min at 4 °C, followed by fixation before acquisition and analyses.

### RNA Extraction and Quantitative RT-PCR.

Total RNA was extracted from lung homogenate and after cDNA conversion detection of mRNA was achieved using specific primers and probes [*SI Appendix*, *Methods*, Applied Biosystems, and (21)].

### Immune Mediator Detection.

Levels of IFN-γ, Granzyme B (both DuoSet ELISA kits from R&D systems), IL-6 (16), and IFN-α (28) present in the BAL fluid were measured by ELISA. Galectin-9 was detected in serum, BAL, and lung homogenate by ELISA (MOFI00838, Assay Genie, IE). Absorbance was determined at 450 nm, on FLUOstar Omega (BMG Labtech) plate readers and analyzed using Mars (BMG Labtech) software.

### ScRNAseq.

FVB/N mice were inoculated i.n. with 1 µg IFN-α (Miltenyi Biotech) 18 h before giving 0.5 × 10^6^ GFP-expressing MMTV-PyMT cells i.v. After 24 h, lung cells were sorted based on CD45^+^ (leukocytes), EpCAM^+^ (epithelial), CD45^−^EpCAM^−^ (mesenchymal), and GFP (PyMT cells) and added at a ratio of 3:3:3:1 as described in *SI Appendix*, *Methods*. Sequencing was performed using a microfluidic chip.

### Bioinformatic Analysis.

Raw reads were initially processed by the Cell Ranger v.2.1.1 pipeline. All subsequent analyses were performed in R v.4.3.3 using the cellrangerRkit packages as described in *SI Appendix*, Methods. Clusters identified using resolution of 0.5 were either assigned manually using specific cell type expression or for immune cells by cross-referencing to the ImmGen dataset (57). fGsea package was used for identification of cancer cell pathway changes using gene expression changes from FindMarkers and cross-referencing to Reactome pathways (58). CellChat was used to identify receptor–ligand interaction changes between the two conditions (59).

### 3D Cell Culture.

5 × 10^3^ MMTV-PyMT GFP^+^ cells were plated into a collagen-coated 96-well Alvetex Scaffold plate (ReproCELL) (44). Next day, lung fibroblasts and lung epithelial cells were isolated 18 h post PBS, IFN-α or RSV exposure as described in *SI Appendix*, *Methods*. 50,000 lung fibroblasts or lung epithelial cells were added to the scaffolds. Results are normalized to the average GFP expression at Day 2 of the PBS condition of each cell type.

### In Vivo Luminescence Imaging.

All experiments were done at The Francis Crick Institute. Exposure to RSV or IFN-α was done in 50 µL. RSV-infected or IFN-α exposed mice were 24 h later inoculated i.v. with 0.5 × 10^6^ MMTV-PyMT actin-luciferase cells, 0.3 × 10^6^ 4T1-luciferase, 0.3 × 10^6^ KP1233-luciferase cells, or 0.4 × 10^6^ RENCA-luciferase cells. In some experiments, Galectin-9 (3 μg/mL) pretreated luciferase-expressing MMTV-PyMT cells were used. At different times post tumor cell injection luminescence signal was quantified using Living Image Software (Perkin Elmer) (60).

### Metastases Burden Analysis.

Mice were killed 28 d after tumor cell injection and metastatic burden was assessed macroscopically and microscopically.

### Incubation of MMTV-PyMT Cells with Galectin-9.

Luciferase-expressing MMTV-PyMT cells were recovered overnight and 1 × 10^7^ cells/mL were incubated with different concentrations of Galectin-9 (Lgasl9; R&D) in PBS for 2 h before use in vivo experiments or plated in 96-well collagen coated plates.

### Live PCLS Staining and Imaging.

PCLS were obtained 18 h after RSV or IFN-α exposure, as previously described (61, 62). PCLS were incubated in complete RPMI (containing 10% FCS, 2 mM L-glutamine, penicillin/streptomycin (100 U/mL); Gibco, Life Technologies) for 15 min at 37 °C. For staining, anti-CD31-PE and anti-Galectin9-Alexa fluor 488 (Biolegend) antibodies were diluted in RPMI media (supplemented with 2% FCS and 1% Pen/strep) and staining was carried out in 350 μL of staining solution per well. Stained PCLS were washed 2× in RPMI media for 5 min with gentle agitation. PCLS were transferred to µ-Plate 24-well IBIDI (Thistle Scientific) containing RPMI media and transferred for imaging. Prior to imaging nuclei were stained using Hoechst (NucBlue™ Live Cell Stain; Molecular Probes, Life Technologies) for 10 min at room temperature. PCLS were imaged using a Leica SP5 MP/FLIM inverted confocal microscope (Leica, Wetzlar, Germany) with a full incubation chamber.

### Statistical Analysis.

Statistical analysis was performed using Prism (GraphPad software) version 9 or 10. Data are presented as the mean ± SEM. As indicated in each figure legend, two tail unpaired Student’s *t* test, two-way ANOVA with mixed-effect analysis or one-way ANOVA was performed. Only *P* values < 0.05, considered statistically significant.

## Supplementary Material

Appendix 01 (PDF)

## Data Availability

The scRNA data are available in Gene Expression Omnibus (GEO) website with the access number GSE303448 (63). All other data are included in the article and/or *SI Appendix*.
