# Supplementary material for: Type I interferons induced upon respiratory viral infection impair lung metastatic initiation
Source: Proc Natl Acad Sci U S A. 2026 Apr 17;123(16):e2412919123. doi: 10.1073/pnas.2412919123 (PMC13099621; doi:10.1073/pnas.2412919123)
Supplement: Supplementary file 1 — Appendix 01 (PDF) [file pnas.2412919123.sapp.pdf]

Supplementary Methods

Mice

The *Myd88/Trif*$^{-/-}$ mice were *Ifna6*$^{gfp/+}$ were bred at Imperial College London. Since *Ifna6* expression was not a primary readout the mice were designated as *Myd88/Trif*$^{-/-}$ mice and compared to *Ifna6*$^{gfp/+}$ wildtype (WT) mice. MMTV-PyMT mice on an FVB/N or C57BL/6J were obtained from The Jackson Laboratory(1). The MMTV-PyMT actin-GFP mice express the green fluorescent protein under the control of the actin promoter were originally a gift from J. Huelsken laboratory (EPFL, Lausanne, Switzerland) and the MMTV-PyMT actin-luciferase mice express firefly luciferase under the control of the actin promoter was a gift from D. Bonnet. All MMTV-PyMT mice were bred at the Francis Crick Institute.

RSV and UV inactivation

Plaque-purified human RSV (originally A2 strain from ATCC, US) was grown in HEp2 cells as previously described(1). UV-inactivated RSV (UV-RSV) was obtained by exposing the virus to UV light for 2 min (UV RSV) in a CX-2000 UV cross-linker.

Experimental lung metastases

MMTV–PyMT, including Luciferase or GFP expressing cells were isolated from breast tumors as previously described(2). Briefly, tumors were mechanically digested with a razor blade and digested with DNase (37.5μl/ml, Roche Diagnostics), Liberase Tm (75μl/ml, Roche Diagnostics), and Liberase Th (75μl/ml, Roche Diagnostics), filtered through a 100μm filter and washed twice in DMEM containing 10% fetal calf serum (FCS). Cells were cultured overnight on collagen coated dishes (30μg/ml PureCol collagen (Advanced Biomatrix), 0.1% bovine serum albumin (BSA, Sigma) and 20 mM HEPES in HBSS (Thermo Fisher Scientific)) in MEM media (DMEM/F12 (Thermo Fisher Scientific) with 2% FCS, 100U/ml penicillin–streptomycin (Thermo Fisher Scientific), 20ng/ml EGF (Thermo Fisher Scientific) and 10μg/ml insulin (Merck Sigma-Aldrich). The following day, cells were washed with PBS followed by a 5 min wash of 1mM EDTA at 37°C. MMTV-PyMT cells were detached with 0.05% Trypsin-EDTA (Gibco) for 7 min at 37°C. Cells were washed with DMEM+10% FCS and frozen in 10% DMSO, 40% FCS, 50% MEM media.

For tail vein injections, cells were thawed and cultured overnight on collagen-coated dishes as previously described. After detachment, cells were washed twice in PBS and filtered through a 100μm filter (pluriSelect).

4T1, 4T1-GFP expressing cells and 4T1-luciferase expressing cells were provided by the Cell Services Unit of The Francis Crick Institute. KP1233-luciferase expressing cells were a kind gift from Dr. Leanne Li (Francis Crick Institute, UK). Cells were grown in DMEM media, supplemented with 10% FCS, 2mM L-glutamine, 100U/ml penicillin, and 100μg/ml streptomycin. Renal cancer cells (RENCA) luciferase expressing cells were provided by the Cell Services Unit of The Francis Crick Institute and grown in RPMI media, supplemented with 10% FCS, 100U/ml penicillin, and 100μg/ml streptomycin. For experimental lung metastases, $0.3x10^6$ 4T1 or 4T1-GFP cells, $0.3x10^6$ 4T1-luciferase or KP1233-luciferase, or $0.4x10^6$ RENCA-luciferase cells were intravenously (i.v.) injected in 100μl of PBS in BALB/c (4T1 and RENCA) and C57BL/6J (KP1233) mice.

Metastatic burden analysis

Mice were sacrificed 28 days after tumor cell injection by a fatal dose of pentobarbital injected i.p.. Lungs were inflated with 1.5ml of PBS and fixed in 10% Formalin (Sigma-Aldrich) for 16h. Macroscopic metastatic burden was quantified by counting nodules on the surface of the lung using the Zeiss SteREO Lumar.V12 stereoscope and is shown as metastatic nodules per lung per mouse. The lungs were then embedded in paraffin blocks. For number of microscopic metastatic nodules and size distribution, three H&E-stained sections of 4μm at least 150μm apart were scanned using the Axio Scan Z1 slide scanner (Zeiss, Germany) and analyzed using the Zen Blue software (Zeiss, Germany). Metastatic tumor burden is shown as fold change to the average of metastatic nodules in the PBS group in each experiment.

Detection of tumor cells by flow cytometry

To quantify tumor cells, lungs were collected at the specified time points after tumor cell injection and digested with Liberase Tm (75μg/ml, Roche Diagnostics), Liberase Th (75μg/ml Roche Diagnostics) and Deoxyribonuclease I (37μg/ml Merck Sigma-Aldrich). in HBSS solution for 30 min at 37°C with 180 rpm agitation. The digested mixture was then passed through a 100μm strainer, washed with 10% FCS HBSS solution and centrifuged for 8 min at 300 *x g*. Cells were then resuspended in Red Blood Cell Lysis Solution (Miltenyi Biotec) for 5 min at room

temperature and then passed through a 40µm strainer. After centrifugation, cells were washed with MACS buffer (0.5% BSA and 2mM EDTA in PBS) and passed through a 20µm strainer-capped tube to generate a single-cell suspension. All recovered cells were stained as described in the flow cytometry section but acquired immediately after staining without fixation.

Isolation of lung immune cells and airway cells

For flow cytometry, lungs were perfused with PBS and collected in complete DMEM (cDMEM; supplemented with 10% FCS, 2mM L-glutamine, 100U/ml penicillin, and 100µg/ml streptomycin). Lungs were processed with a gentle MACS dissociator (Miltenyi Biotech) according to manufacturer's protocol and digested with Collagenase D (1mg/ml; Roche) and DNase I (30µg/ml; Invitrogen) for 1h shaking at 37 °C and processed again in a gentle MACS dissociator. Red blood cells were lysed using an ACK buffer (0.15M NH_4Cl, 1.0 mM $KHCO_3$, 0.1mM Na_2EDTA). Cells were re-suspended in PBS and filtered through a 100µM cell strainer (Greiner Bi One) and the absolute number of recovered cells was quantified by Trypan Blue (Thermo Fisher Scientific) exclusion of dead cells.

Bronchoalveolar lavage (BAL) was performed by flushing 1ml of PBS with 0.5mM EDTA through the trachea 3 times. The BAL was centrifuged, the supernatant was stored at −80 °C and the cells were treated with ACK buffer to remove red blood cells. Total airway cell number was determined by Trypan Blue (Thermo Fisher Scientific) exclusion of dead cells. Lung and airway cells were further analyzed by flow cytometry.

Flow cytometry

For immune cell characterization, 2.5×10^6 lung cells or all recovered BAL cells were incubated with a purified rat IgG2b anti–mouse CD16/CD32 receptor antibody (BD) for 20 min at 4°C in FACS buffer (PBS supplemented with 1% BSA and 0.05mM EDTA). For surface staining, cells were stained in PBS with fixable live-dead Aqua dye (Invitrogen) and with different fluorochrome-conjugated antibodies (see Supp. Table 1) for 25 min at 4°C. Following staining, cells were fixed with 100µl 1% paraformaldehyde or Cytofix™ Fixation Buffer (BD) for 20 min at 4°C and stored in FACS buffer. Analysis was performed on a BD LSR Fortessa, by acquiring $2.5x10^5$ single, live $CD45^+$ cells. Data were analyzed with FlowJo software (Tree Star). Total cell populations were

quantified as the whole lung count x (proportion of lung tissue sampled) x (%CD45$^+$ cell of live cells) × (% of population of interest out of CD45$^+$ cells).

RNA extraction and quantitative RT-PCR

For RNA extractions, lung lobes were snap-frozen in liquid nitrogen and stored at −80°C. Lungs were homogenized using a TissueLyser LT (Qiagen) and total RNA was extracted from the lung tissue supernatant using RNeasy Mini kit (Qiagen) including a DNase digestion step. For RNA extraction of MMTV-PyMT, cells were lysed in 350µl of RLT buffer and total RNA was extracted using RNeasy Mini kit (Qiagen) including a DNase digestion step. RNA yield was determined by NanoDrop (Thermo Scientific). Conversion to cDNA of 1 or 2 µg of RNA was performed using High Capacity RNA-to-cDNA kit (Applied Biosystems). Real time PCR was performed using Quantitect Probe PCR Master Mix (Qiagen) in the 7500 Fast Real-Time PCR System (Applied Biosystems). Detection of mRNA of *Gapdh* (encoding glyceraldehyde-3-phosphate dehydrogenase), *Cxcl1, Ccl2, Il1b, Ifna5, Cxcl10, Mx1*, *Il6, Acta2, Vim, Cdh1 and Cdh2* was achieved using specific primers and probes (all Applied Biosystems). Relative mRNA expression of the genes of interest is shown as $2^{-\Delta CT}$. Quantitative PCRs for *Ifnb, Ifnl, Oas1, Rsad2 (Viperin)* and *Eif2ak2 (Pkr)* and RSV L gene was performed using primers and probes previously described(1). Analysis was performed using 7500 Fast System SDS Software (Applied Biosystems). Number of copies of the gene of interest are shown as copy number per ug of RNA and was calculated using a plasmid DNA standard curve for each gene and normalized to *Gapdh*, as previously described(3). Analysis was performed using 7500 Fast System SDS Software (Applied Biosystems).

Antibody-mediated immune cells depletion.

- Antibody mediated neutrophil depletion

FVB/N mice were intraperitoneally (i.p.) treated with 150µg in 100µl of anti-Ly6G (clone 1A8, Assay Genie, IE) or IgG isotype control (clone Y13/238, Cell services, the Francis Crick Institute) a day before RSV infection and every second day until day 4 post infection.

- Antibody mediated monocyte depletion

C57BL/6J mice were treated with 20µg of anti-CCR2 (clone MC21(4)) or isotype-matched control rat IgG2b (Assay Genie, IE) in 100µl i.p. 6h before RSV infection and daily until day 5 p.i..

- Antibody mediated NK cell depletion

C57BL/6J mice were treated with 100µg of anti-NK1.1 (clone PK136, eBioscience) or isotype mouse IgG2a (clone C1.18.4, BioXcell) in 100µl i.n. 24h before RSV infection. Mice were treated with 200µg in 200µl of anti-NK1.1 or isotype control i.p. the day before infection and daily until day 4 post infection.

- Antibody mediated T cell depletion

C57BL/6J mice were exposed to PBS or RSV i.n.. Depletion of T cells was performed by i.p. administration of 150µg of anti-CD4 (clone GK1.5, Assay Genie, IE) and 150µg of anti-CD8 (clone YTS 169) from the day of infection until day 6 p.i. every alternate day. Isotype-matched control of 300µg rat IgG2b (Assay Genie, IE) was performed following the same regime.

scRNAseq

FVB/N mice were inoculated with one dose of 1µg IFN-α (Miltenyi) 18h before giving 0.5x10^6 GFP-expressing MMTV-PyMT cells i.v.. After 24h, the lungs, pooled from each condition, were digested using Liberase and stained for cell surface markers. The single cell suspension was divided in two. One portion of the sample remained untouched whereas the rest of the sample was depleted of $CD45^+$ cells via MACS prior to cell sorting via FACS, samples were fixed for 16h using a 4% formaldehyde fixative solution, as described in the Demonstrated Protocols CG000478 and CG000553 and using Chromium Next GEM Single Cell Fixed RNA Sample Preparation Kit (10x Genomics PN-1000414), prior to sorting (BD Influx). A total of 5 mice exposed to PBS and 20 mice exposed to IFN-α were used. Cells were sorted based on $CD45^+$ (leukocytes), $EpCAM^+$ (epithelial), $CD45^-EpCAM^-$ (mesenchymal) and GFP (PyMT cells) and added to the final sample at a ratio of 3:3:3:1. Cells were pooled from 3 independent experiments. Quench buffer (10x Genomics, PN-2000516) was added to the sample for storage at -80°C. Gene expression was measured using barcoded probe pairs designed to hybridize to mRNA specifically. Using a microfluidic chip, the fixed and probe-hybridized single cell suspensions were partitioned into nanolitre-scale Gel Beads-in-emulsion (GEMs). A pool of ~737,000 10x GEM Barcodes was sampled separately to index the contents of each partition. Inside the GEMs, probes were ligated and the 10x GEM Barcode was added, and all ligated probes within a GEM share a common 10x GEM Barcode. Barcoded and ligated probes were then pre-amplified in bulk, after which gene expression libraries were generated (User Guide: CG000527, Chromium Fixed RNA Profiling Reagent Kits for Multiplexed Samples) and sequenced (NovaSeq 6000. Sequencing read configuration: 28-10-10-90).

Bioinformatic Analysis

Raw reads were initially processed by the Cell Ranger v.2.1.1 pipeline, which deconvolved reads to their cell of origin using the UMI tags, aligned these to the mm10 transcriptome using STAR (v.2.5.1b) and reported cell-specific gene expression count estimates. Genes were 'expressed' if the estimated (log_{10}) count was at least 0.1. Primary filtering was then performed by removing from consideration: genes expressed in fewer than 3 cells; cells expressing fewer than 200 genes; cells for which the total yield (that is, sum of expression across all genes) was more than two standard deviations from the mean across all cells in that sample; and cells for which mitochondrial genes made up greater than 4% of all expressed genes. Seurat V5(5) was used to perform UMAP and for nearest-neighbour analysis on the integrated datasets (rPCA). Clusters identified using resolution of 0.5 were either assigned manually using specific cell type expression or for immune cells by cross-referencing to the ImmGen dataset(6). fGSEA package was used for identification of cancer cell pathway changes using gene expression changes from FindMarkers and cross-referencing to Reactome pathways(7). CellChat was used to identify receptor-ligand interaction changes between the two conditions(8).

3D cell culture

Lungs were harvested 18h post PBS, IFN-α or RSV exposure and enzymatically digested, as described in the section 'Detection of tumor cells by flow cytometry. Cells were then labelled with CD45 and CD31 MicroBeads (Miltenyi Biotec) for magnetic depletion of immune and endothelial cells according to manufacturer's protocol and the remaining cells were stained with a fluorescent-conjugated EpCAM antibody for sorting via FACS(9, 10). The purity of lung fibroblasts ($CD45^-CD31^-EpCAM^-$) or lung epithelial cells ($CD45^-CD31^-EpCAM^+$) were above 95%.

In vivo luminescence imaging

At different times post tumor cell injection luminescence signal (photons/second) was quantified using Living Image Software (Perkin Elmer) by selecting a ROI of a set size positioned over the thorax of each animal. *Ex vivo* bioluminescence intensity was measured to assess metastatic burden at 28 days post cell injection(11). To measure luciferase activity, at different times post tumor cells injection, animals were administered 100μl of 30mg/ml D-Luciferin (Xenogen) i.p..

After 12 min the animals were anaesthetized with inhaled isoflurane. The anaesthetized animals were then imaged using IVIS Spectrum (Perkin Elmer)(11).

Lung homogenate

A lung lobe was snap-frozen and then thawed in 350μl of PBS containing protease and phosphatase inhibitor cocktail (PPC1010, Sigma, 1:500). The tissue was homogenized using a tissue lyser for 30 seconds at 50Hz twice with a minute rest on ice. The homogenate was span for 10min at 10000 *x g* at 4°C. The supernatant volume was adjusted to 50mg of tissue/ml. Protein concentration was measured using a Pierce™ BCA Protein Assay Kit (Thermo Scientific).

Immunohistochemistry

Tissue was fixed for 24h in 10% NBF before processing to wax using a Tissue-Tek VIP® 6 AI processor. 3μm FFPE sections were cut and baked for 1h at 60°C before staining. Single IHC staining was performed on the Leica Bond Rx automated stainer. Galectin-9 (Abcam, ab216479) was stained at 1:50 dilution. BOND Polymer Refine Detection kit (Leica, DS9800) was used, including hydrogen peroxidase, anti-rabbit polymer, DAB and haematoxylin counterstain. Antigen retrieval with ER2 at 95˚C for 20min was performed prior to primary antibody incubation. Slides were counterstained with Haematoxylin and cover slipped using TissueTek Prisma plus automated stainer.

Confocal imaging of PCLS

PCLS were imaged using a Leica SP5 MP/FLIM inverted confocal microscope (Leica, Wetzlar, Germany) with a full incubation chamber. Hoechst was excited with a 405nm diode laser, Galectin-9 was excited using a 488nm argon laser, CD31 were excited using a 543nm HeNe laser. A tile scan was performed with images acquired using 1024x1024-pixel format with a z-stack step size of 0.5micron. Images were acquired with a 16-bit imaging depth with a line averaging of 4. Maximum projection images were generated, and 3D visualisation and export were performed on Imaris v10.2 (Oxford Instruments). Images were acquired using the Leica LAS software.

Viability of MMTV-PyMT cells after incubation with Galectin-9

Viability was determined at 24 and 48h after Galectin-9 exposure using the CellTiter-Glo Luminescent Cell Viability Assay (Promega) following manufacturer's protocol. To quantify viability, control wells were considered 100% viable. The average luminescent values were obtained from 3-5 technical replicates, each from 3 different luciferase-expressing MMTV-PyMT preparations.

References:

1. M. Goritzka, *et al.*, Alveolar macrophage-derived type I interferons orchestrate innate immunity to RSV through recruitment of antiviral monocytes. *J Exp Med* **212**, 699–714 (2015).

2. I. Malanchi, *et al.*, Interactions between cancer stem cells and their niche govern metastatic colonization. *Nature* **481**, 85–89 (2012).

3. M. Goritzka, *et al.*, Alpha/beta interferon receptor signaling amplifies early proinflammatory cytokine production in the lung during respiratory syncytial virus infection. *J Virol* **88**, 6128–6136 (2014).

4. M. Mack, *et al.*, Expression and characterization of the chemokine receptors CCR2 and CCR5 in mice. *J Immunol* **166**, 4697–4704 (2001).

5. Y. Hao, *et al.*, Dictionary learning for integrative, multimodal and scalable single-cell analysis. *Nat Biotechnol* **42**, 293–304 (2024).

6. The Immunological Genome Project Consortium, *et al.*, The Immunological Genome Project: networks of gene expression in immune cells. *Nat Immunol* **9**, 1091–1094 (2008).

7. M. Milacic, *et al.*, The Reactome Pathway Knowledgebase 2024. *Nucleic Acids Research* **52**, D672–D678 (2024).

8. S. Jin, M. V. Plikus, Q. Nie, CellChat for systematic analysis of cell–cell communication from single-cell transcriptomics. *Nat Protoc* (2024). https://doi.org/10.1038/s41596-024-01045-4.

9. L. Ombrato, *et al.*, Metastatic-niche labelling reveals parenchymal cells with stem features. *Nature* **572**, 603–608 (2019).

10. F. S. Rodrigues, *et al.*, Bidirectional activation of stem-like programs between metastatic cancer and alveolar type 2 cells within the niche. *Dev Cell* S1534-5807(24)00338–1 (2024). https://doi.org/10.1016/j.devcel.2024.05.020.

11. S. K. Wculek, I. Malanchi, Neutrophils support lung colonization of metastasis-initiating breast cancer cells. *Nature* **528**, 413–417 (2015).

Supplementary Material

Supp. Table 1. Antibodies used for flow cytometry, for gating strategies refer to Supp. Fig.2, 3, 5 ,6, 7, 8, 10 and 11.

**Target**	**Clone**	**Fluorochrome**	**Company**	**Working concentration**
CD45	30-F11	BV605	Biolegend	0.25 µg/ml
CD45	30-F11	PerCP-Cy5.5	Biolegend	0.25 µg/ml
Siglec F	E50-2440	BV786	BD Bioscience	0.5 µg/ml
Ly6G	1A8	Alexa Fluor 488	Biolegend	5 µg/ml
CD64	X54-5/7.1	APC	Biolegend	1 µg/ml
CD64	X54-5/7.1	PE	Biolegend	1 µg/ml
CD11c	HL3	V450	BD Bioscience	1 µg/ml
CD11b	M1/70	PE-Cy7	eBioscience	0.5 µg/ml
CD3	145-2C11	APC-efluor780	eBioscience	2 µg/ml
CD19	1D3	APC	Biolegend	1 µg/ml
CD3	17A2	Alexa Fluor 700	eBioscience	2 µg/ml
CD8	53-6.7	eFluor780	eBioscience	0.5 µg/ml
CD4	GK1.5	PE	eBioscience	0.5 µg/ml
CD4	RM4-5	APC	BD Bioscience	0.5 µg/ml
CD69	H1.2F3	BUV737	BD Bioscience	1 µg/ml
CD49b	DX5	PE	Biolegend	1 µg/ml
Ly6C	12HK1.4	BV711	eBioscience	0.5 µg/ml
EpCAM	G8.8	APCFire750	eBioscience	2 µg/ml
EpCAM	G8.8	APC	eBioscience	2 µg/ml
PD-1	BV605	29F.1A12	Biolegend	2 µg/ml

Supp. Table 2. Markers used for the annotations in the single cell RNA sequencing data set, refer to Fig. 3E.

Annotation	Markers
Artery Endothelial	*"Pecam1", "Cdh5", "Gja5", "Bmx"*
Vein Endothelial	*"Pecam1", "Cdh5", "Ackr1", "Cpe"*
aCAP Endothelial	*"Car4", "Ednrb", "Fibin", "Tbx2", "Cdkn2b", "Rprml", "Chst1", "Apln"*
gCAP Endothelial	*"Gpihbp1", "Plvap", "Cd93", "Ptprb", "Tek", "Cxcl12", "Aplnr"*
Lymphatic Endothelial	*"Prox1", "Pdpn", "Mmrn1", "Maf", "Cp", "Prox1", "Thy1", "Ccl21a", "Ptprc"*
AT2 Epithelial	*"Sftpb", "Sftpc", "Sftpd", "Muc1", "Etv5"*
AT1 Epithelial	*"Ager", "Pdpn"*
Club Epithelial	*"Cyp2f2", "Scgb3a2", "Scgb1a1"*
Ciliated Epithelial	*"Ccdc153", "Dynlrb2", "Sec14l3", "Tmem212", "Fam183b", "Tppp3", "Rsph1", "Ccdc39", "Riiad1", "Epcam", "Foxj1", "Tubb1", "Tp73", "Ccdc78"*
Mesothelial Stromal	*"Msln", "Upk3b", "Wt1"*
Pericytes	*"Cspg4", "Trpc6", "Pdgfrb"*
Smooth Muscle Fibroblasts	*"Cnn1", "Acta2", "Tagln", "Notch3", "Lgr5", "Des", "Lgr6",*
Myofibroblasts	*"Col1a1", "Pdgfra", "Eln", "Acta2"*
Lipofibroblasts	*"Inmt","Gpx3","Ogn","Mfap4","Mgp","Gsn", "Limch1","Pcolce2", "Tcf21", "Wnt2"*
Immune Cells	Reference Immgen dataset

Supplementary data

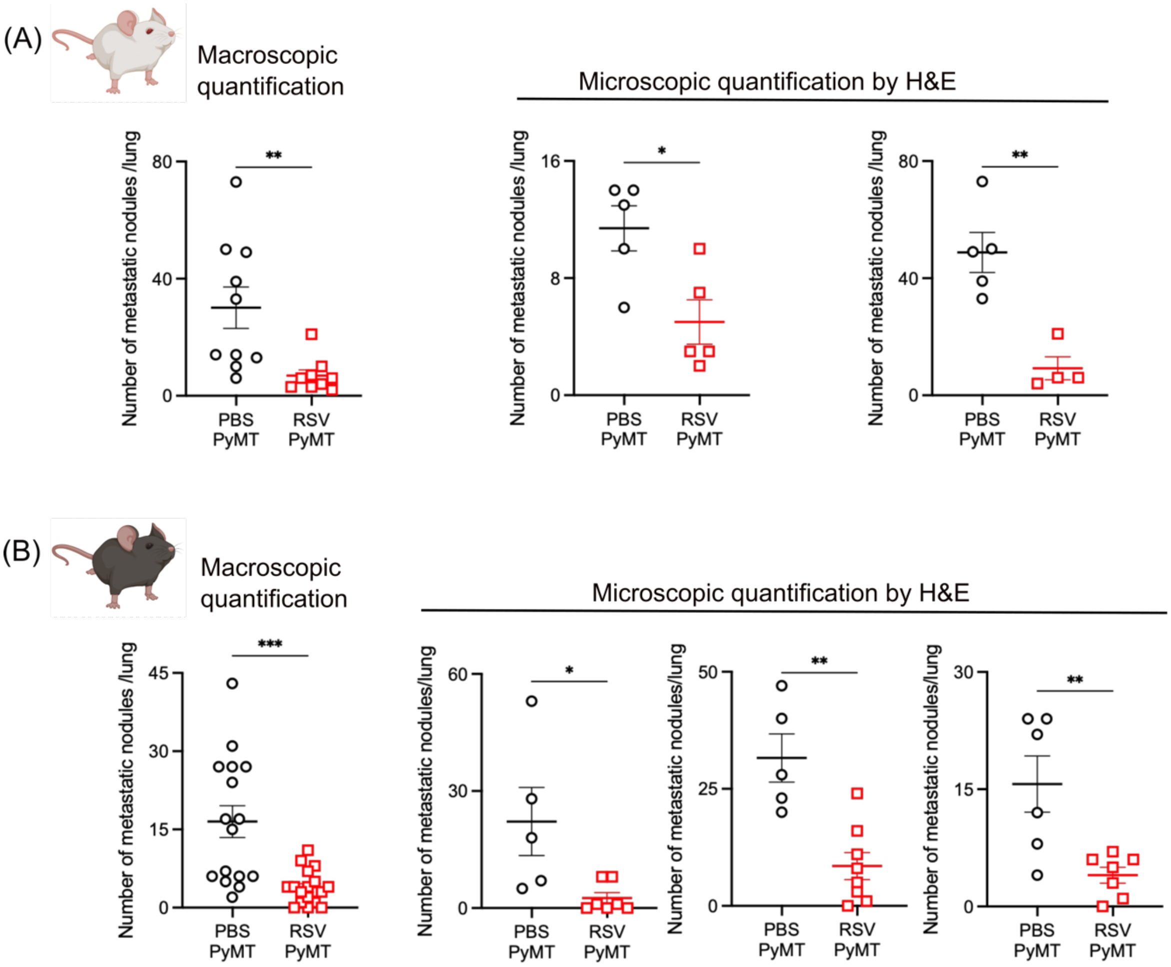

Supp. Fig. 1. Macroscopic and microscopic quantification of metastatic MMTV-PyMT nodules in mock or RSV infected mice. (A) FVB/N or (B) C57BL/6J mice received MMTV-PyMT cells i.v. a day after RSV (RSV PyMT) or mock-infection (PBS PyMT) and the lungs were analyzed for tumors 28 days later. Gross macroscopic quantification of lung metastatic nodules was performed. Also, microscopic quantification performed by quantifying nodules in H&E-stained lung sections is shown for each independent experiment. These data were normalized and pooled in Fig. 1. Data shown for macroscopic count are pooled from two independent experiments n=10 for PBS and 9 for RSV infected FVB/N mice and from three independent experiments n=16 for PBS and n=23 for RSV infected C57BL/6J mice. All data are shown as mean+/-SEM. Student's *t* test statistical analysis was performed; *p<0.05, **p<0.01, ***p<0.0005.

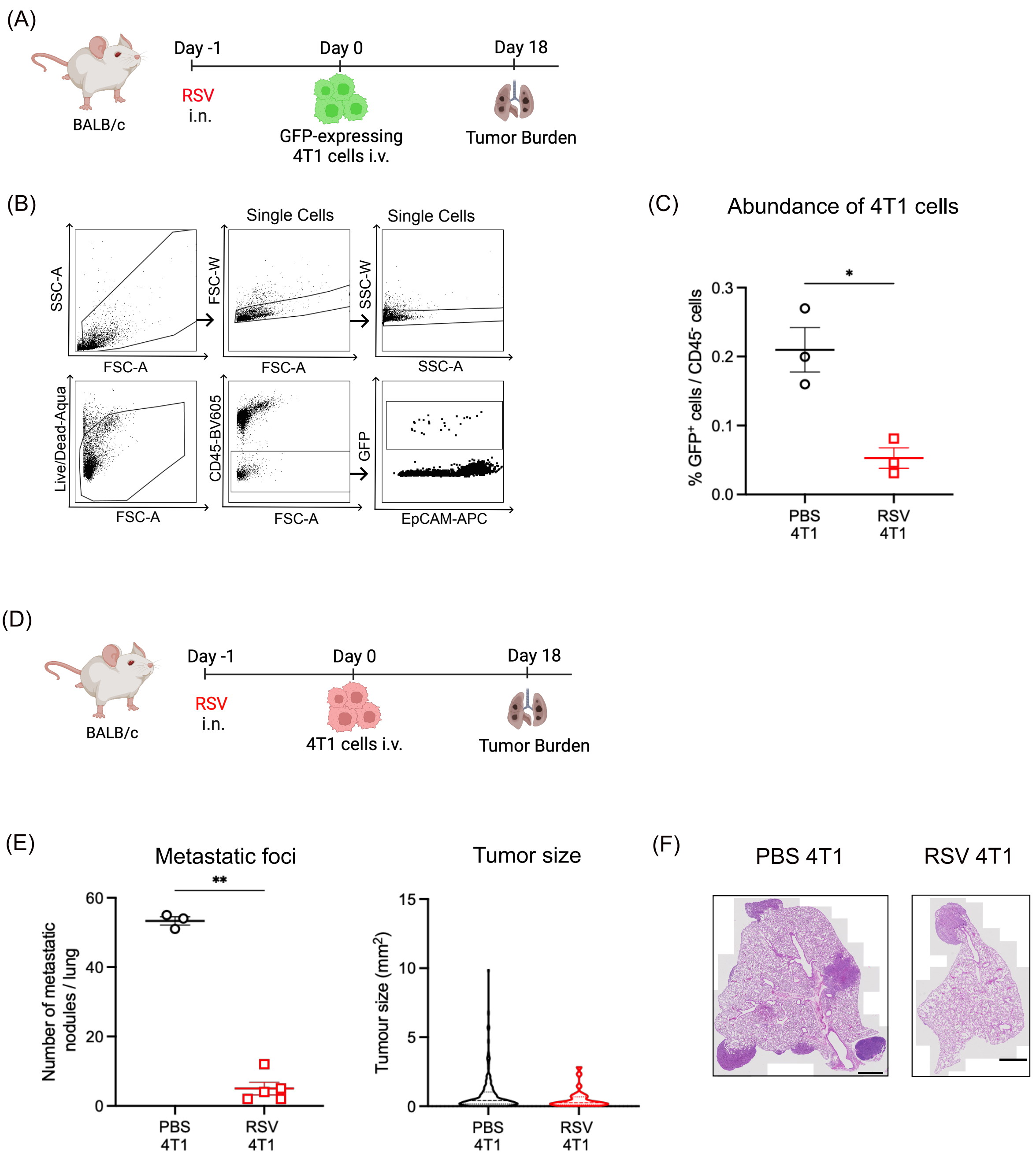

Supp. Fig. 2. RSV infection impairs 4T1 experimental lung metastasis. (A) BALB/c mice were infected i.n. with RSV or mock infected (PBS). A day later mice were injected i.v. with $1x10^5$ GFP expressing 4T1 cells. Abundance of tumor cells in the lungs was assessed a week later by flow cytometry. (B) Gating strategy and (C) percentage of GFP^+ 4T1 cells in the lungs of infected or control mice. The same set up was used with unlabeled 4T1 cells. (E) Number of metastatic nodules and their size was assessed by histological analysis after 18 days. (F) Representative H&E-stained section of the lung, scale bar 1000μm. Data from one experiment shown as mean +/- SEM, with (C) 3 mice per group and (E) 3 mice in the control group and 5 mice in the infected group. Student's *t* test was performed, and only statistically significant differences are shown; *p<0.05, **p<0.01

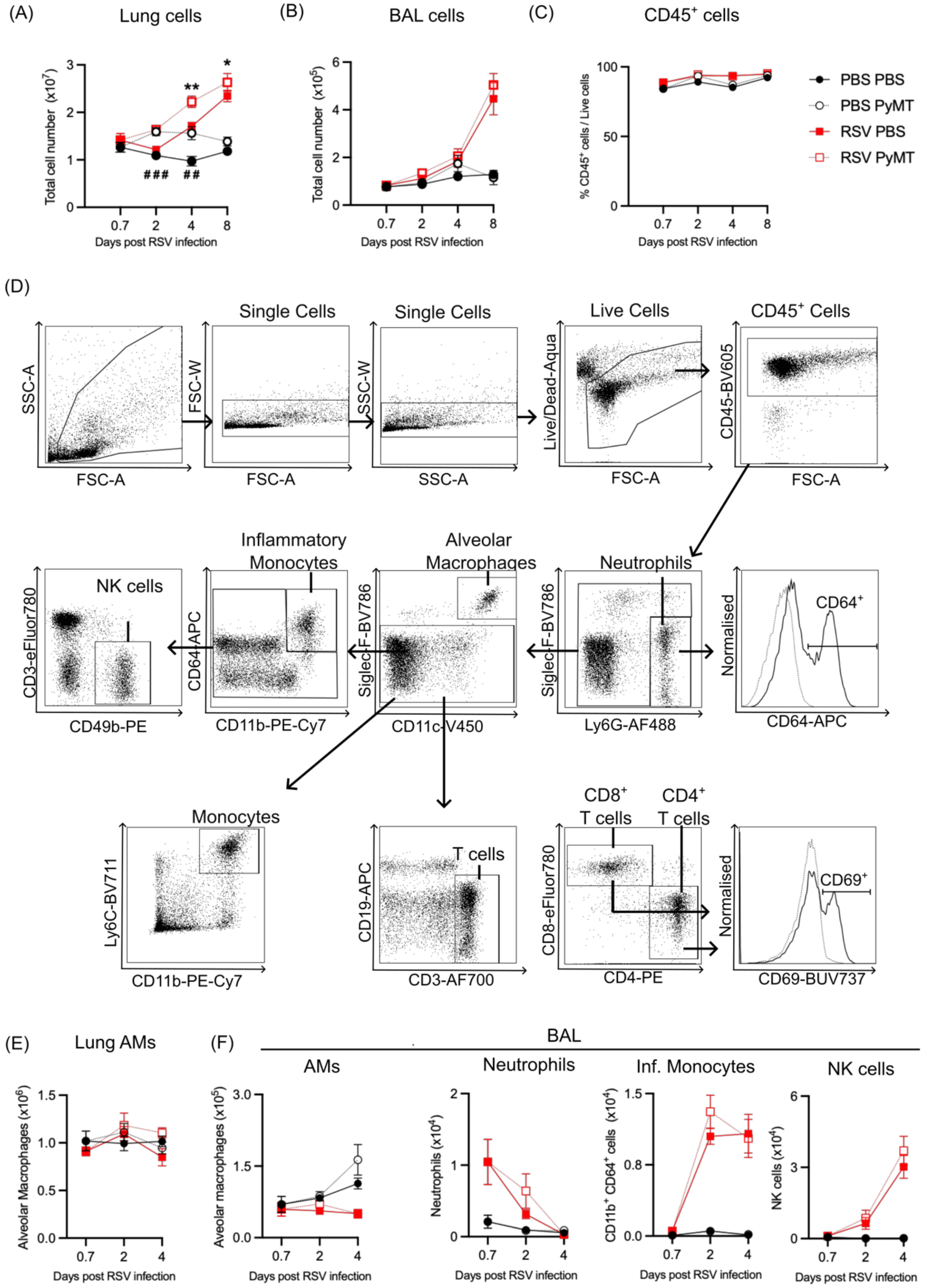
(A)
Lung cells
Total cell number (x10^7)
0
1
2
3
**
*
###
##
0.7
2
4
8
Days post RSV infection
(B)
BAL cells
Total cell number (x10^5)
0
2
4
6
0.7
2
4
8
Days post RSV infection
(C)
CD45+ cells
% CD45+ cells / Live cells
0
50
100
0.7
2
4
8
Days post RSV infection
PBS PBS
PBS PyMT
RSV PBS
RSV PyMT
(D)
SSC-A
FSC-A
Single Cells
FSC-W
FSC-A
Single Cells
SSC-W
SSC-A
Live Cells
Live/Dead-Aqua
FSC-A
CD45+ Cells
CD45-BV605
FSC-A
NK cells
CD3-eFluor780
CD49b-PE
Inflammatory Monocytes
CD64-APC
CD11b-PE-Cy7
Alveolar Macrophages
SiglecF-BV786
CD11c-V450
Neutrophils
SiglecF-BV786
Ly6G-AF488
CD64+
Normalised
CD64-APC
Monocytes
Ly6C-BV711
CD11b-PE-Cy7
T cells
CD19-APC
CD3-AF700
CD8+ T cells
CD4+ T cells
CD8-eFluor780
CD4-PE
CD69+
Normalised
CD69-BUV737
(E)
Lung AMs
Alveolar Macrophages (x10^6)
0.0
0.5
1.0
1.5
0.7
2
4
Days post RSV infection
(F)
BAL
AMs
Aveolar macrophages (x10^5)
0.0
1.5
3.0
0.7
2
4
Days post RSV infection
Neutrophils
Neutrophils (x10^4)
0
1
2
0.7
2
4
Days post RSV infection
Inf. Monocytes
CD11b+ CD64+ cells (x10^4)
0.0
0.8
1.5
0.7
2
4
Days post RSV infection
NK cells
NK cells (x10^4)
0
3
6
0.7
2
4
Days post RSV infection

Supp. Fig. 3. Tumor cells injected a day after RSV infection do not alter the anti-viral immune response. FVB/N mice were intranasally (i.n.) infected with RSV or mock-infected (PBS). A day later, $3x10^5$ MMTV-PyMT cells were intravenously (i.v.) injected (PBS PyMT and RSV PyMT). Cells in the lungs (A) and airways (BAL; B) were enumerated. (C) Percentage of $CD45^+$ cells recovered from the lungs quantified by flow cytometry. (D) Gating strategy to identify cell populations in lungs and airway during RSV infection in the presence of MMTV-PyMT cells. Shown are representative flow cytometry plots from lung cells harvested 18h or 8 days (T cells) post RSV infection. Flow cytometry analysis depicting the frequency of live $CD45^+$ cells after excluding debris, doublets and dead cells and downstream gating strategy used to define neutrophils, activated neutrophils ($CD64^+$), alveolar macrophages (AMs), inflammatory monocytes, gated as $CD11b^+Ly6C^+$ or $CD11b^+CD64^+$, NK cells and $CD8^+$ and $CD4^+$ T cells. T cell activation was determined by CD69 expression. Numbers of (E) lung AMs and (F) AMs, neutrophils, inflammatory monocytes (gated as $CD11b^+$ $CD64^+$) and NK cells present the airways at different times post infection were analyzed by flow cytometry. Data are pooled from two independent experiments presented as the mean+/-SEM; for day 0.7; n= 6 mice in the PBS group and n= 7 in the RSV group, for day 2; n= 9 mice per group. Days 4 and 8; n= 8 mice per group. One-way ANOVA was performed to compare the PBS and PyMT mice (#) and the RSV and RSV PyMT mice (*) at each time point. Only statistically significant differences are shown; *p<0.05, **p<0.01, ##p<0.01, ###p<0.001.

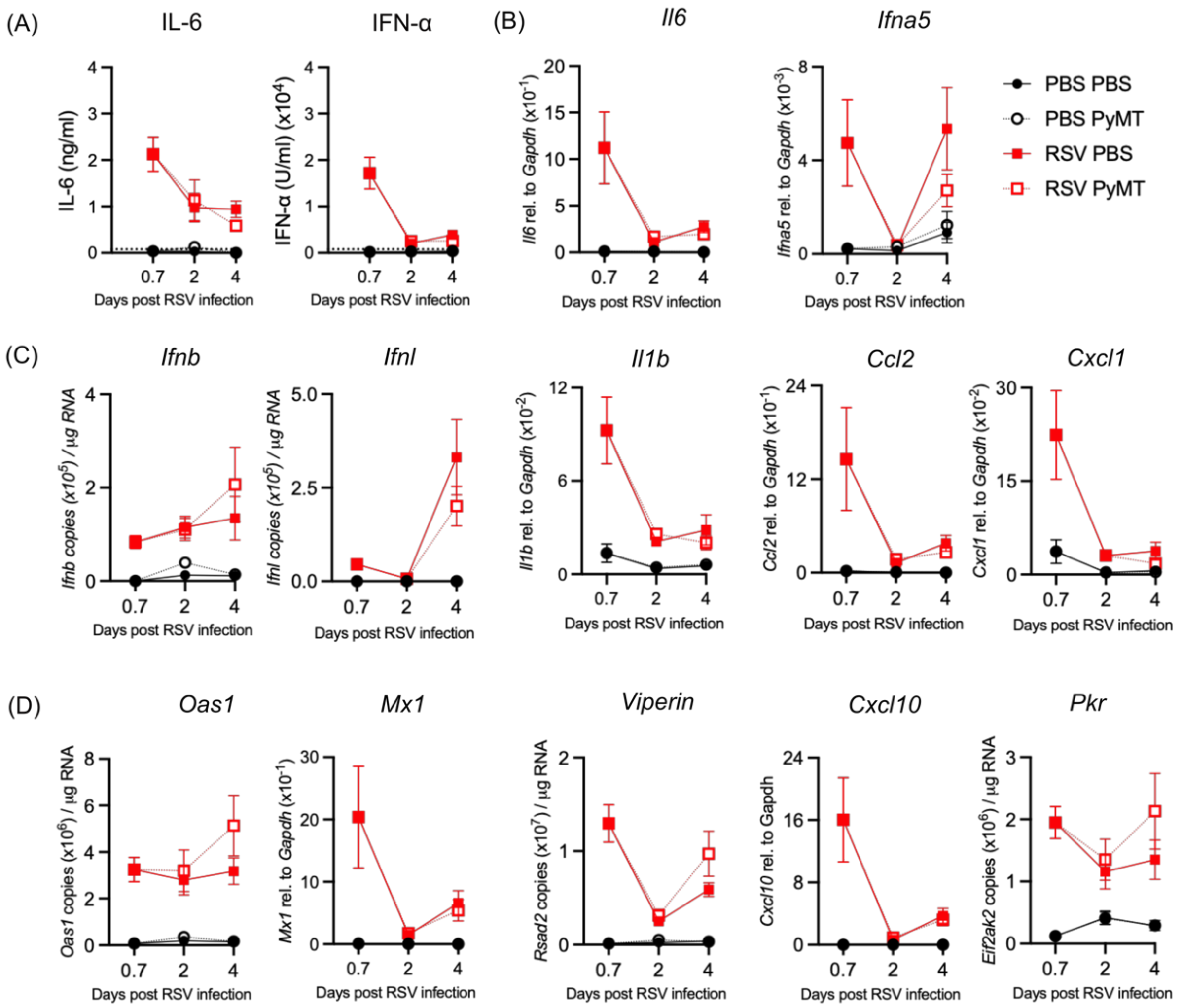

Supp. Fig. 4. Immune responses in lungs and airways during RSV infection in the presence of MMTV-PyMT cells. FVB/N mice were infected i.n. with RSV or mock infected (PBS). A day later 3x10^5 MMTV-PyMT cells (PyMT) or PBS were injected i.v.. (A) BAL was obtained at different time points after infection and the levels of IL-6 and IFN-α were quantified by ELISA. Expression of (B) *Il6, Ifna5* (C) *Ifnb, Ifnl, Il1b, Ccl2,* and *Cxcl1 and* (D) the interferon stimulated genes *Oas1, Mx1, Viperin, Cxcl10* and *Pkr*, were quantified in RNA from lungs by RT-qPCR. Data for day 0.7 are pooled from two independent experiments presented as the mean+/-SEM of 9 mice per group. Data for days 2 and 4 are pooled from two independent experiments with 8 mice per group. One-way ANOVA was performed to compare the infected groups followed by Tukey's post hoc test at each time point. No statistically significant differences were detected.

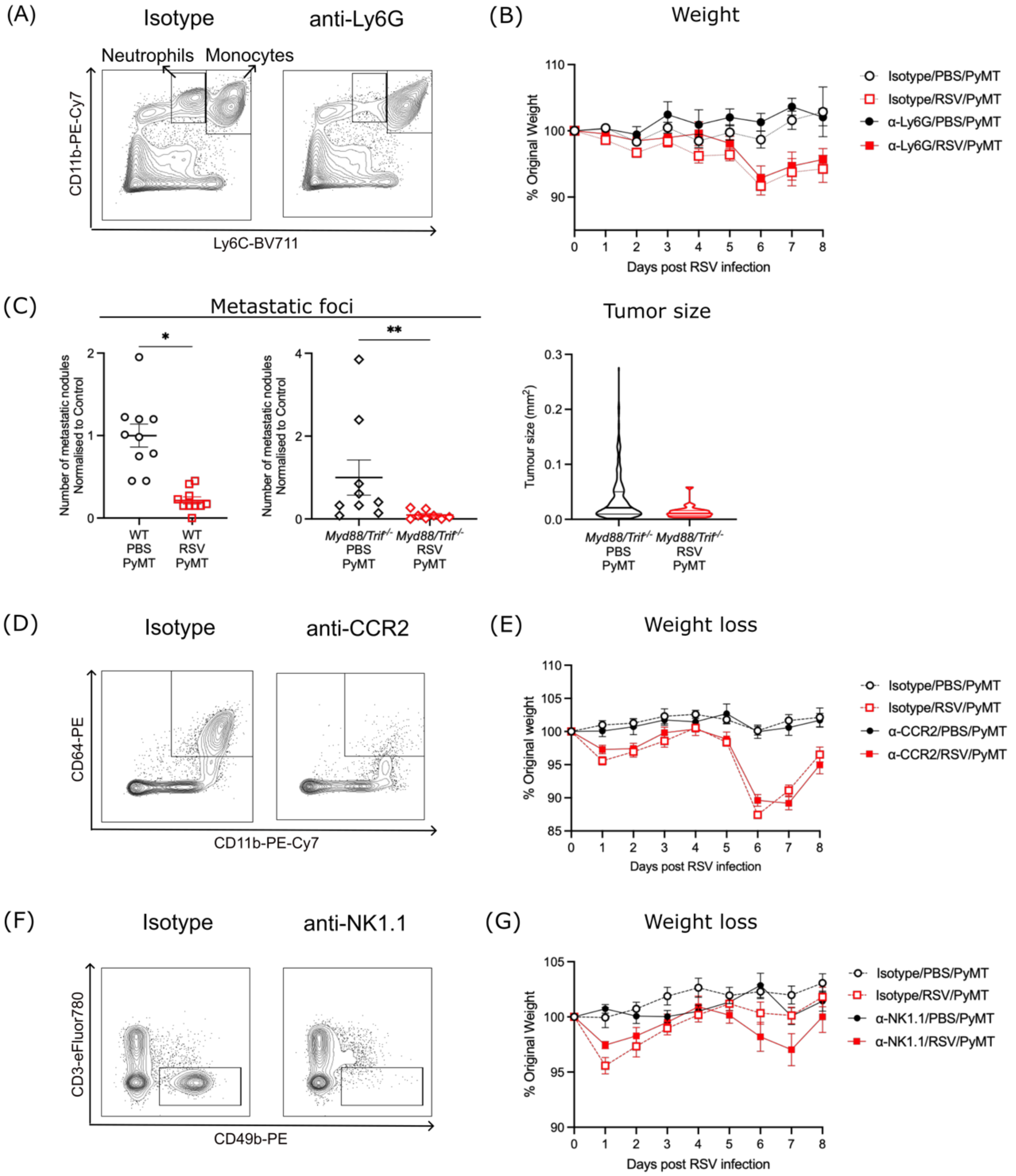

Supp. Fig. 5. Reduced number of metastatic nodules is not due to RSV-induced recruitment of neutrophils, monocytes or NK cells. (A) FVB/N mice were treated i.p. with anti-Ly6G or isotype control every second day from the day prior to infection. Representative flow cytometry of CD11b^{+} Ly6C^{+} lung cells gated on live, CD45^{+}, SiglecF^{-} cells on day 2 p.i.. (B) Disease severity after RSV infection was follow by weight loss. The data are pooled from two independent experiments presented as the mean+/-SEM of 9 mice in the PBS group and 8 or 10 RSV infected

mice treated with isotype or anti-Ly6G, respectively. (C) *Myd88/Trif*$^{-/-}$ or wildtype (WT) mice were exposed i.n. to PBS or RSV at day -1 and then injected with MMTV-PyMT cells (PyMT) i.v. on day 0. Lungs were analyzed for tumor burden after 28 days. Number of metastatic nodules and tumor size were quantified 28 days post tumor inoculation by H&E staining. Data are pooled from two independent experiments and shown as mean+/-SEM of n=10 in each wildtype group and n=9 for the *Myd88/Trif*$^{-/-}$ PBS group and n=8 for the *Myd88/Trif*$^{-/-}$ RSV group. Student's *t* test statistical analysis was performed *p<0.05, **p<0.01. (D) Monocytes were depleted in C57BL/6J mice using anti-CCR2 from the day of infection. Representative dot plot of lung CD11b^{+} CD64^{+} cells, gated on live, CD45^{+}, SiglecF^{-}, Ly6G^{-} cells at day 2 p.i.. (E) Disease severity after RSV infection was followed by weight loss until day 8 p.i.. The data are pooled from two independent experiments presented as the mean+/-SEM of 6 mice/isotype treated groups and 8 mice in the anti-CCR2 groups. (F) C57BL/6J mice were depleted of NK cells following i.p. and i.n. the day prior to infection, and then i.p. administration of anti-NK1.1 antibody daily from day -1 to day 3. Mice were infected with RSV or mock-infected (PBS) at day -1 and inoculated with tumor cells at day 0. Depletion of NK cells in the lung was analyzed by flow cytometry at day 4 p.i., gated on live, CD45^{+}, SiglecF^{-}, Ly6G^{-}, CD3^{-}, CD49b^{+} lung cells. (G) Disease severity after RSV infection was followed by weight loss. The data are pooled from two independent experiments presented as the mean+/-SEM of 9 mice/isotype treated groups and 10 mice/anti-NK1.1 groups. (B, E, G) A two-way ANOVA, mixed-effect analysis was performed to compare weight loss after infection followed by Tukey's post hoc test, no significant differences were detected.

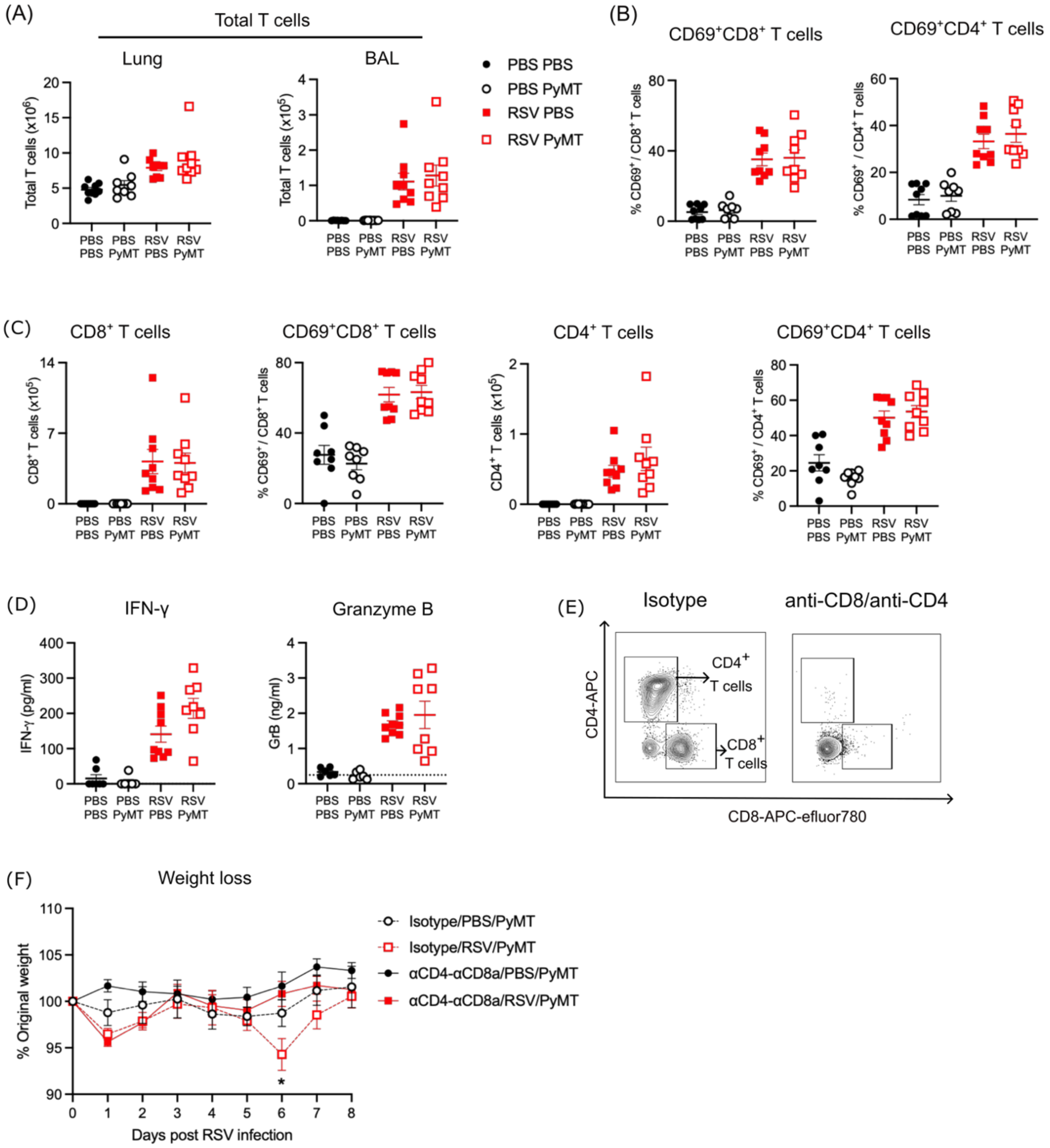

Supp. Fig. 6. T cell responses in lungs and airways during RSV infection in the presence of MMTV-PyMT cells. FVB/N mice were infected i.n. with RSV or mock-infected with PBS. A day later, $3x10^5$ MMTV-PyMT cells were injected i.v.. (B) Number of T cells ($CD3^+$) recovered from lungs and BAL 8 days p.i.. (B) T cell activation measured as $CD69^+$ or $PD1^+$ T cells were quantified in (B) lungs or (C) BAL 8 days p.i., following the gating strategy shown in Supp. Fig. 3D. (D) At 8 days p.i., IFN-γ and Granzyme-B in BAL were quantified by ELISA. Data are pooled from two independent experiments, with n=8 for uninfected groups and n=9 for RSV infected groups.

Student's *t* test was performed to compare the two RSV infected groups, with no differences detected. (E) $CD4^+$ and $CD8^+$ T cells were depleted during RSV infection using the respective antibodies or isotype control every second day, from the day of infection until day 6 p.i.. Representative dot plot showing $CD4^+$ and $CD8^+$ T cells from lungs at day 7 p.i., gated on live, $CD45^+$, $SiglecF^-$, $Ly6G^-$, $CD3^+$ cells. (F) Weight loss after RSV infection was followed daily until day 8 p.i.. Data are representative from two independent experiments presented as the mean+/-SEM of 9 mice per group in the PBS group and 8 or 10 mice in RSV infected mice treated with isotype or anti-CD4 and anti-CD8, respectively. A two-way ANOVA, mixed-effect analysis was performed to compare weight loss after infection followed by Tukey's post hoc test, only significant differences are shown, *p<0.05.

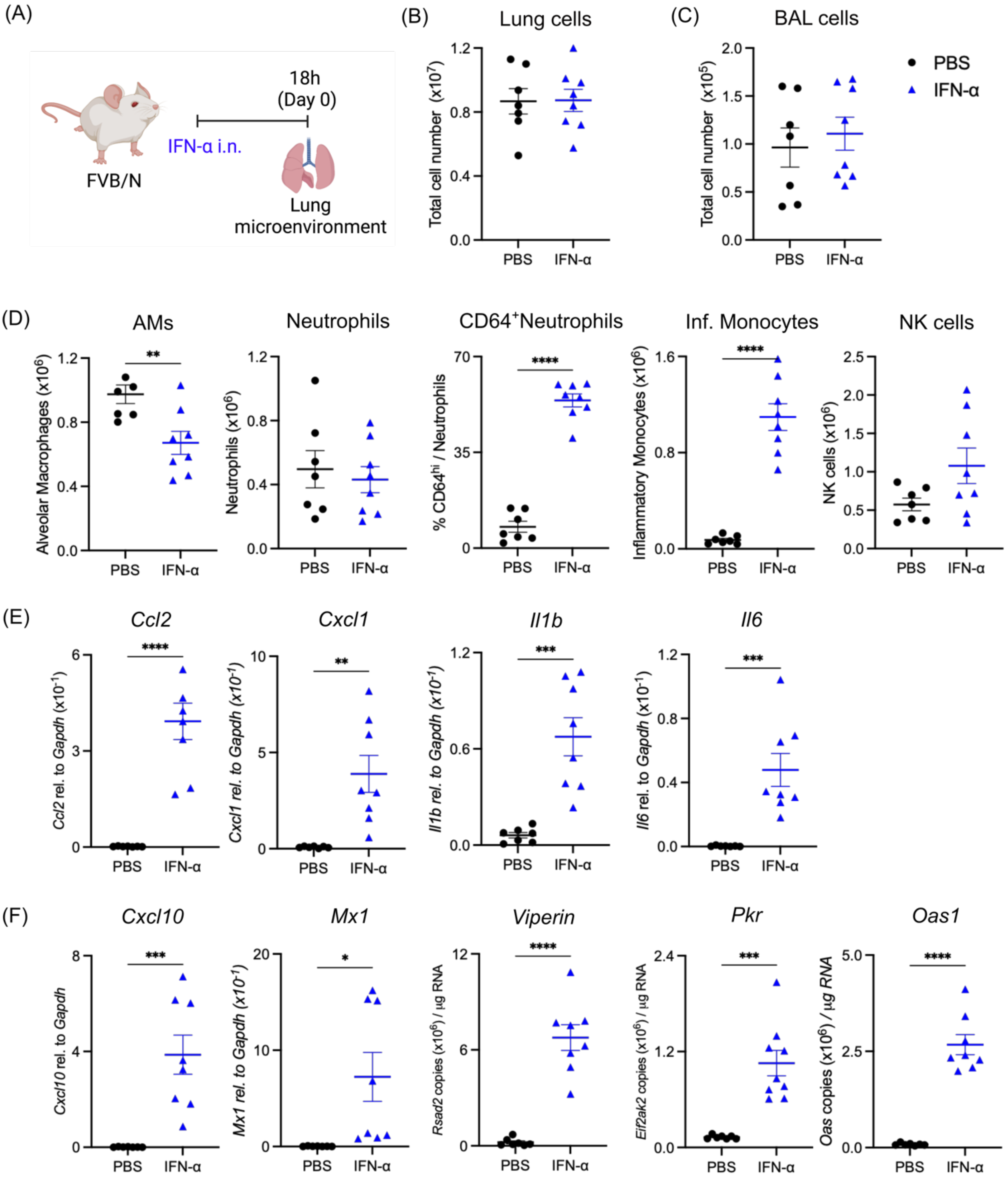

Supp. Fig. 7. Intranasally administered recombinant IFN-α induce transient changes in the lung microenvironment. FVB/N mice were treated i.n. with 1µg of IFN-α and 18h later lungs and BAL were obtained. (A) Schematic representation of the treatment regime. At 18h, total number of (B) lung cells and (C) BAL cells were quantified. (D) AMs, neutrophils, CD64⁺ neutrophils, inflammatory (Inf) monocytes and NK cells were quantified after PBS or IFN-α exposure. (E)

Relative gene expression of *Ccl2, Cxcl1, Il1b, Il6* and (F) relative gene expression of gene copy number of the interferon stimulated genes *Mx1, Viperin, Pkr, Cxcl10* and *Oas1* were quantified and normalized to housekeeping gene *Gapdh* or quantified using a standard curve. PBS data are also shown as PBS day 0.7 in Fig. 2. Data shown are pooled from two independent experiments with n=6 for PBS and n=7 for IFN-α +/-SEM. Student's *t* test analysis was performed. Only statistically significant differences are shown; *p<0.05, **p<0.01, ***p<0.005, ****p<0.0001.

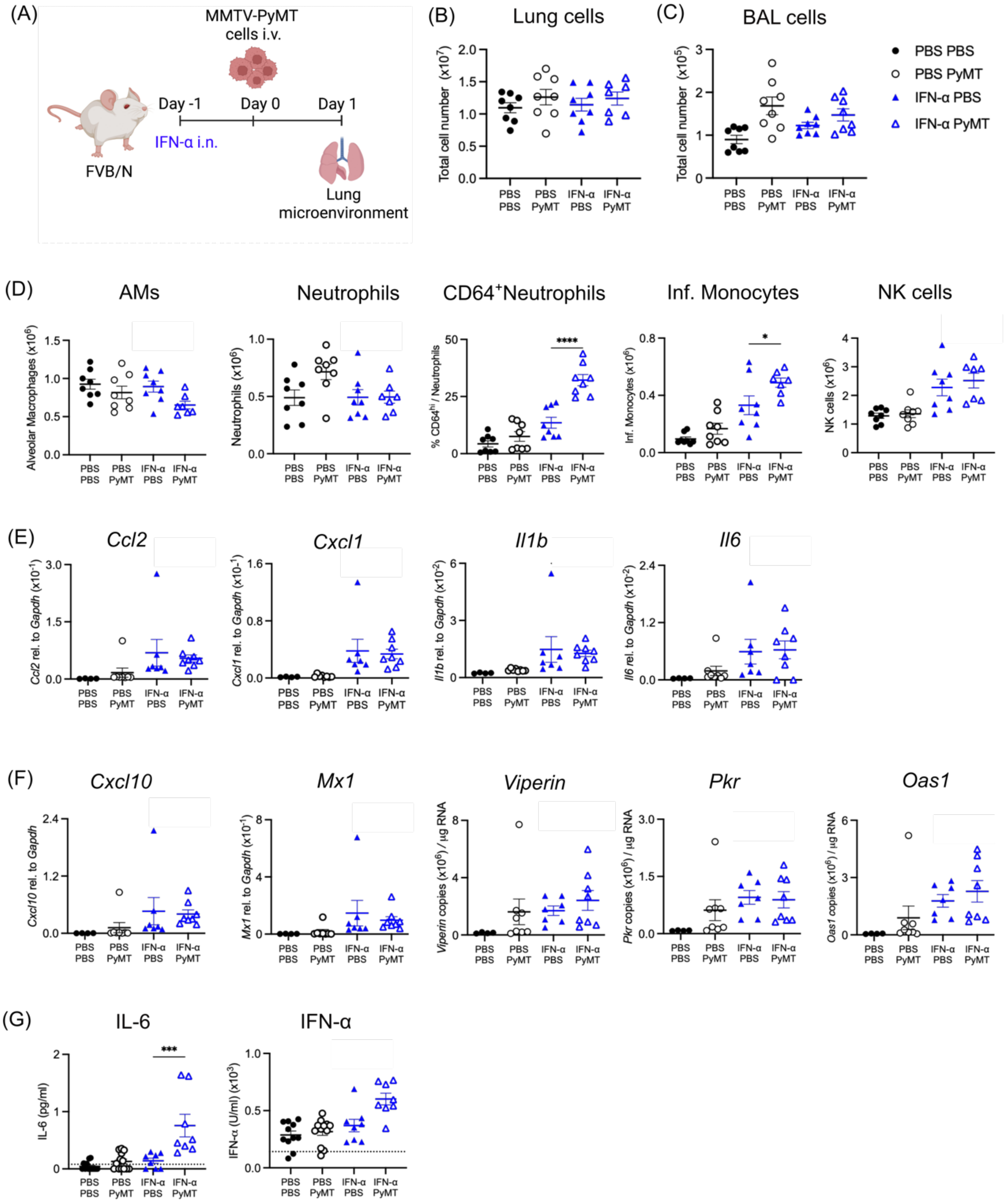

Supp. Fig. 8. Presence of MMTV-PyMT cells in the lungs do not change the response induced by IFN-α. FVB/N mice were treated i.n. with 1μg of IFN-α at day -1, injected with tumor cells at day 0 and lungs and BAL were harvested 18h later. (A) Schematic representation of the treatment regime. At 18h post MMTV-PyMT cell administration, total number of (B) lung cells and (C) BAL cells were quantified. (D) Innate immune cells in the lungs were quantified by flow

cytometry. (E) Relative gene expression of *Ccl2, Cxcl1, Il1b, Il6* and (F) relative gene expression or gene copy number of the interferon stimulated genes *Cxcl10, Mx1, Viperin, Pkr and Oas1* were quantified and normalized to housekeeping gene *Gapdh*. Number of copies were calculated using a plasmid standard curve for *Viperin*, *Pkr* and *Oas1*. (G) Concentration of IL-6 and IFN-α was quantified in BAL fluid, dotted line shows detection limit. PBS data are also shown as PBS day 2 in Fig. 2. Data shown are representative from two independent experiments with n=8 +/- SEM. One-way ANOVA with Tukey's post hoc test was performed to compare the IFN-α exposed groups. Only statistically significant differences are shown; *p<0.05; ***p<0.005; ****p<0.0001.

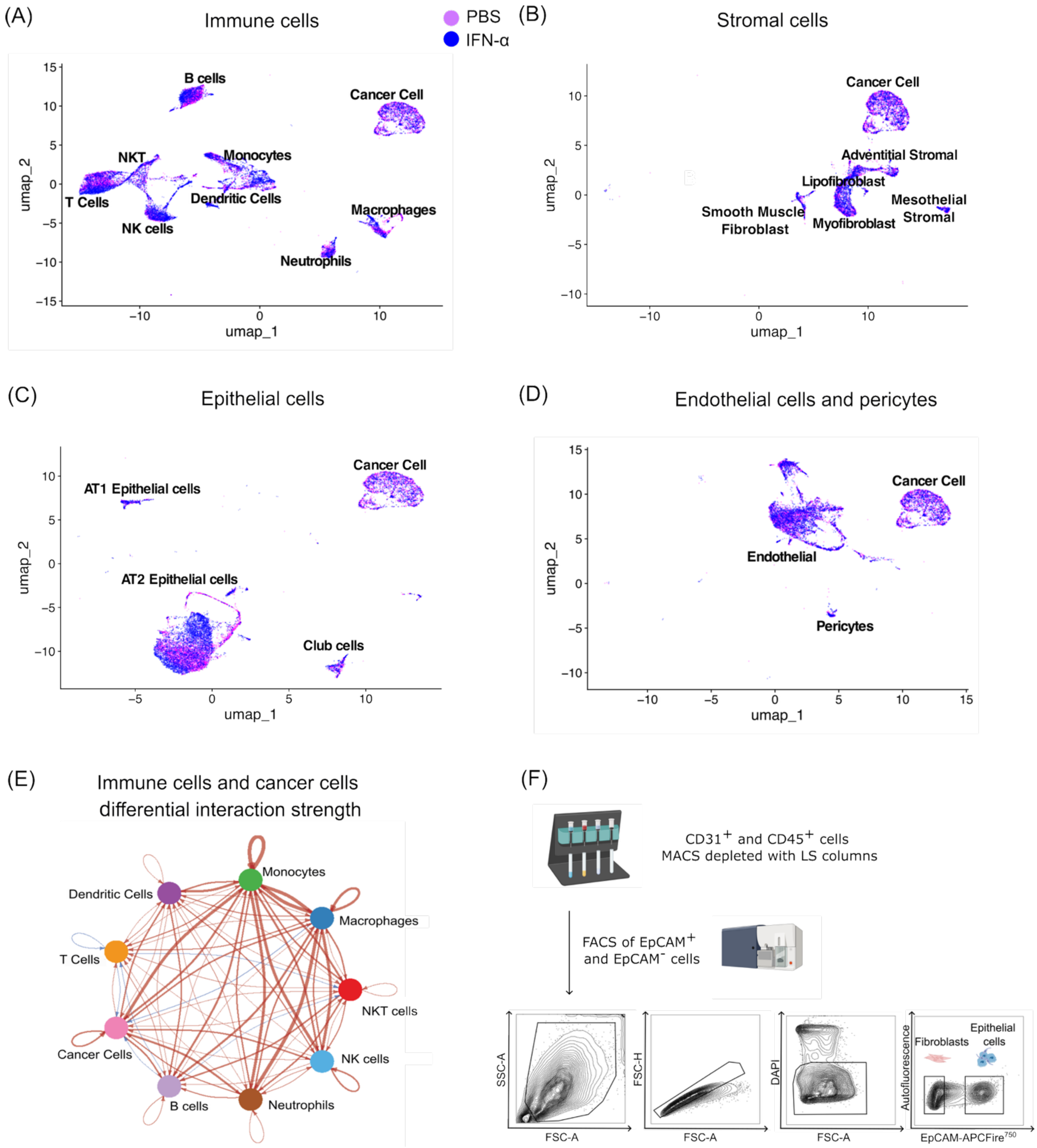

Supp. Fig. 9. Effect of IFN-α on the lung and the cancer cells. scRNAseq data analysis from samples obtained as shown in Figure 4D. Overlapped UMAP from IFN-α and mock-treated showing (A) immune cell clusters, (B) stromal cell clusters (C) epithelial cell clusters and (D) endothelial cell and pericyte clusters as well as cancer cells in the lung. (E) Circle plot visualizing the directionality of Differential Interaction Score of Cancer Cells with immune cell components based on Secreted ligands and Receptor interactions examined by CellChat analysis. (F) C57BL/6J mice were infected i.n. with RSV, exposed to 1μg of IFN-α or mock treated (PBS). Lungs were harvested 18h later and were subjected to liberase digestion to obtain a single cell suspension.

To enrich the populations of interest, lung CD45^{+} and CD31^{+} cells were depleted using LS columns. The remaining cells were stained for viability and EpCAM for further sorting via FACS. Shown are representative flow cytometry plots from lung cells from a mock infected mouse.

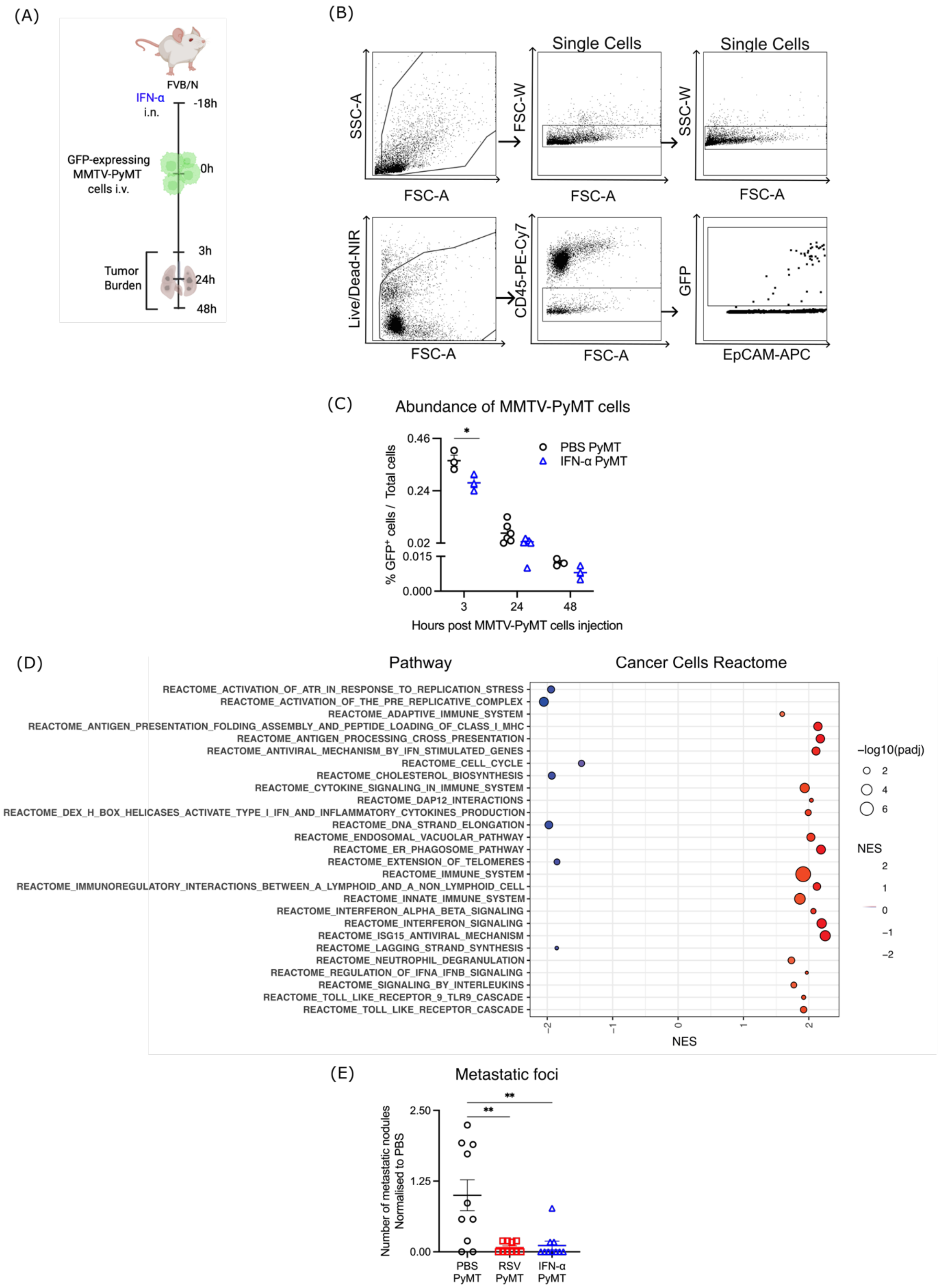

Supp. Fig. 10. Intranasal exposure to IFN-α impairs homing of tumor cells to the lungs. (A) FVB/N mice were intranasally exposed to 1μg of IFN-α or PBS. GFP-expressing MMTV-PyMT cells were injected i.v. 18h later. (B and C) Abundance of tumor cells in the lungs was quantified by

flow cytometry, as shown in the gating strategy, at 3, 24 or 48h after tumor cell injection. (D GSEA analysis of Cancer Cell Reactome pathways only showing the top statistically significant pathways with a Normalized Enrichment Score (NES) >1 or <-1. (E) Number of metastatic nodules in FVB/N mice exposed to IFN-α or RSV a day prior to MMTV-PyMT luciferase expressing cell injection quantified at 28 days by H&E staining of 3 levels of each lobe, total number of metastatic tumors was normalized to the average of the uninfected group in each independent experiment. Data are pooled from two independent experiments, with n=10 mice per group. One-way ANOVA test followed by Tukey's post hoc test was performed to compare all groups. Only statistically significant differences are shown; *p<0.05, **p<0.01.

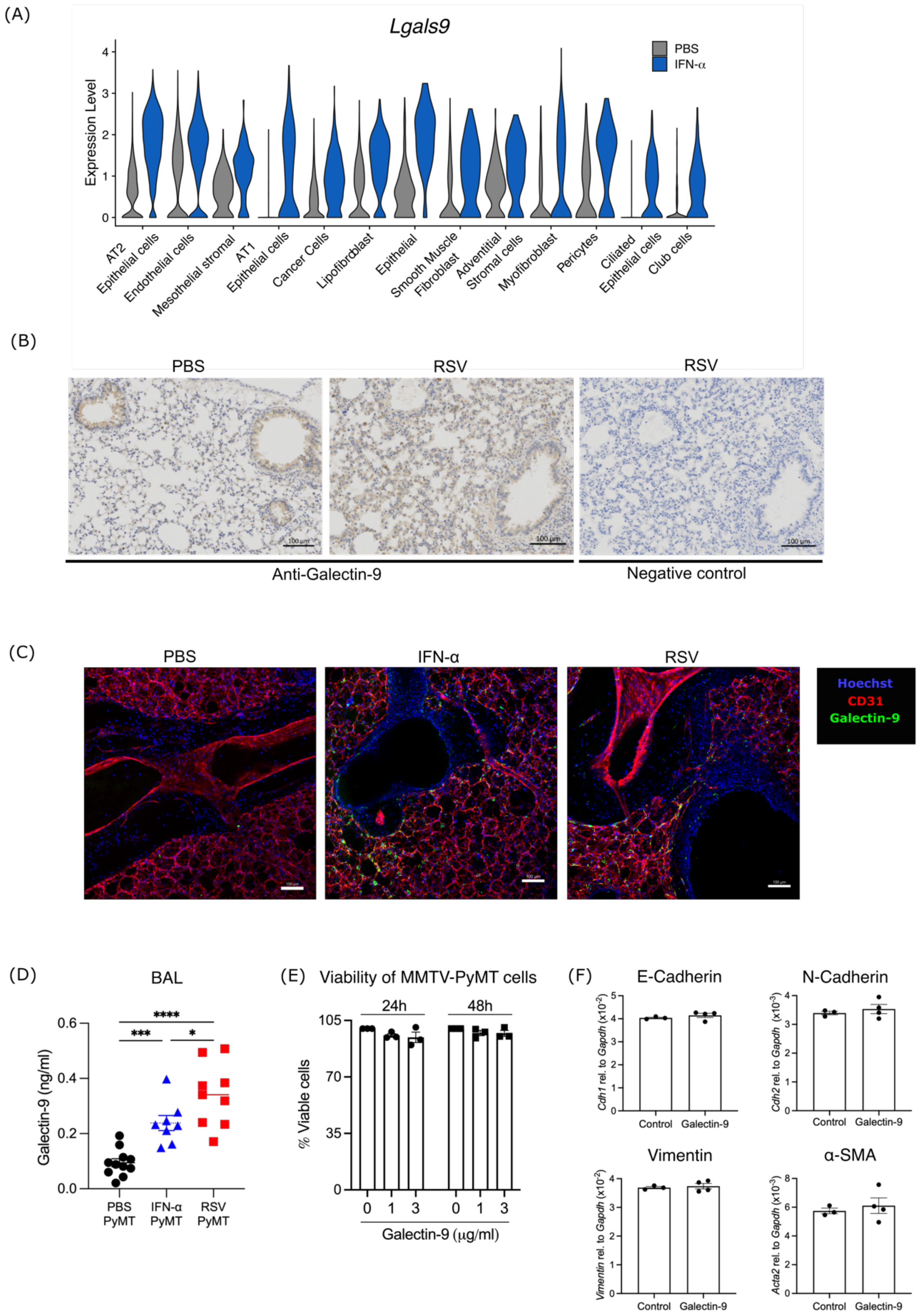

Supp. Fig. 11. IFN-α induces expression of Galectin-9 in most resident pulmonary cells. (A) Violin plot of Galectin-9 (*Lgals9*) expression in non-immune cell clusters, split by treatment (PBS, grey or IFN-α, blue). (B) Galectin-9 expression was detected by immunohistochemistry in lung sections (C) or by immunofluorescence staining of PCLS, 18h after intranasal infection with RSV.

(D) Levels of Galectin-9 in the airways (BAL) of mice exposed to IFN-α or infected with RSV, then injected with MMTV-PyMT cells and sampled 24h later. The samples used are the same as the 2 d.p.i. in Figure 2. (E) MMTV-PyMT cells were incubated with different concentrations of Galectin-9 for 2h. The cells were then cultured in 96-well collagen-coated plates. Viability was assessed using CellTiter-Glo Luminescent Cell Viability Assay after 24 and 48h. (F) MMTV-PyMT cells were incubated with 3μg/ml of Galectin-9 for 2h and then cultured for 24h in collagen-coated 6-well plates. Levels of mRNA of *Cdh1* (E-Cadherin), *Cdh2* (N-Cadherin), *Vimentin* and *Acta2* (α-SMA) were quantified by qPCR. (E-F) The data shown are representative of two experiments.
